# A dileucine motif in TMEM163 is essential for its binding with both AP-3 and BLOC-1 complex

**DOI:** 10.1016/j.jbc.2026.111451

**Published:** 2026-04-14

**Authors:** Zhuang Qi, Yefeng Yuan, Wei Li

**Affiliations:** 1 Laboratory for Genetics of Birth Defects, Beijing Pediatric Research Institute, MOE Key Laboratory of Major Diseases in Children, Genetics and Birth Defects Control Center, National Center for Children's Health, Beijing Children's Hospital, Capital Medical University, Beijing, China; 2 Henan Key Laboratory of Genetic and Developmental Disorders, Institute of Children’s Health, Henan Academy of Medical Sciences, Zhengzhou, China; 3 Zhengzhou Hospital of Beijing Children’s Hospital, Zhengzhou, China

**Keywords:** dileucine motif, Hermansky-Pudlak syndrome, HPACs, platelet dense granules, TMEM163

## Abstract

Defects in Hermansky-Pudlak syndrome protein associated complexes impair the biogenesis of lysosome-related organelles (*e.g.* platelet dense granule). Transmembrane protein 163 (TMEM163), a zinc transporter, is drastically reduced in platelets of AP-3-, BLOC-1-, and BLOC-2-deficient Hermansky-Pudlak syndrome mice and patients, but the mechanistic basis is unclear. We here found that TMEM163 was reduced and degraded primarily *via* the proteasome in a variety of deficient MEG-01 cells of endosomal trafficking complexes (AP-1, AP-2, AP-3, BLOC-1, BLOC-2). A conserved N-terminal acidic dileucine motif (LEDRGL^69^L^70^) in TMEM163 is essential for interactions with BLOC-1 and AP-3 but dispensable for binding with AP-1, AP-2 and BLOC-2. Mutation of this motif led to TMEM163 accumulation in the plasma membrane. Loss of either BLOC-1 or AP-3 enhanced TMEM163 bound to the other and exhibited differential abnormal endo-lysosomal localization, suggesting competitive binding during TMEM163 trafficking. Collectively, our findings established TMEM163 as a cargo protein sequentially sorted by AP-3 and BLOC-1 *via* a shared dileucine-based sorting signal, which is essential for its proper trafficking to platelet dense granules, uncovering a unique mechanism in Hermansky-Pudlak syndrome protein associated complex cargo sorting and lysosome-related organelle biogenesis.

Lysosome-related organelles (LROs) represent a specialized class of organelles that share functional and morphological similarities with lysosomes. The biogenesis of LROs critically depends on specific protein complexes, including biogenesis of lysosome-related organelles complexes one to 3 (BLOC-1, -2, -3), adapter protein complex 3 (AP-3), and homotypic fusion and protein sorting complex (1). This process involves intricate molecular mechanisms in endolysosomal trafficking pathways. Genetic defects disrupting these pathways lead to Hermansky-Pudlak syndrome (HPS), a group of disorders characterized by LRO defects. Currently, 11 HPS subtypes have been identified. Specifically, mutations in *HPS7, HPS8, HPS9* and *HPS11* lead to BLOC-1 deficiency; mutations in *HPS3, HPS5* and *HPS6* cause BLOC-2 deficiency; mutations in *HPS1* and *HPS4* cause BLOC-3 deficiency; mutations in *HPS2* and *HPS10* cause AP-3 deficiency. Notably, these HPS protein associated complexes (HPACs) are tightly bound heteromers in which loss of one subunit often destabilizes the others (2, 3).

HPS manifests with diverse pathological changes due to altered protein trafficking to tissue-specific LROs, such as albinism and bleeding tendency. Mutations in the *AP3B1/HPS2* gene impair AP-3 complex function, preventing the proper trafficking of ATP8A1 to lamellar bodies (one type of LRO) in alveolar type II (AT2) cells through recognition of a C-terminal dileucine-based signal. Consequently, ATP8A1 accumulates in early endosomes, contributing to lung fibrosis pathogenesis (4). Similarly, the maturation of melanosomes, another LRO, relies on membrane transport pathways involving AP-3 and BLOC-1 (5). Central to this trafficking machinery are the acidic dileucine (LL) motifs ([D/E]XXXL[L/I]) in cargo proteins, which are recognized by heterotetrameric adapter protein complexes AP-1, AP-2, and AP-3. While AP-1 and AP-3 direct cargoes from Golgi-to-endosome or plasma membrane (PM) and from endosome-to-lysosome transport, AP-2 mediates endocytosis from the PM.

The binding site for these motifs is structurally and functionally conserved across AP complexes, consisting of one of γ/α/δ/ε/ζ, one β, one μ, and one σ subunit (6). Despite this conservation, binding affinity and specificity vary depending on the specific signal sequence and AP complex, allowing for selective cargo recognition (6, 7, 8, 9, 10). For instance, OCA2 utilizes dileucine-dependent binding to both AP-1 and AP-3 (11), while tyrosinase also interacts with AP-1 and AP-3, and TYRP1 interacts specifically with AP-1 but not AP-3 (12). Low-density lipoprotein receptor-related protein 9 binds to AP-1 and AP-2 *via* the EDEPLL motif at the carboxyl terminus of its cytosolic tail (13). It seems that different LRO cargo proteins may utilize different sorting machineries mediated by APs during trafficking.

Originally identified in rat brain synaptosomes as synaptic vesicle protein 31 (14), transmembrane protein 163 (TMEM163) is a zinc transporter predicted to possess six transmembrane domains. TMEM163 transcripts are widely expressed in human and rodent tissues, including brain, lung, pancreas, kidney, ovary, testis, and platelets (15, 16, 17, 18, 19). TMEM163 localizes to multiple cellular compartments, including the plasma membrane, lysosomes, early endosomes, synaptic vesicles, and other vesicular structures, as demonstrated by heterologous expression in HEK293T cells (15, 16, 18), immunocytochemistry and subcellular fractionation in PC-12 cells (20). Specifically, TMEM163 localizes to platelet dense granules (DGs) and insulin granules, implicating its roles in DG biogenesis (17) and insulin granule packaging/secretion (16). TMEM163 as a dimer binds and transports zinc in a proton-dependent manner (21). TMEM163 also engages in membrane protein interactions: TRPML1 (Mucolipin-1) with its loss causing Mucolipidosis type IV (18), and ATP-gated P2X receptor (22), implicating its role in regulating organelle homeostasis.

Previously, we found that TMEM163 expression was significantly reduced in platelets from mice and patients harboring mutations in BLOC-1, BLOC-2, and AP-3, but unaffected in BLOC-3 or homotypic fusion and protein sorting complex deficiency. Furthermore, we demonstrated that BLOC-1 specifically regulates TMEM163 transport to DGs, and that loss of TMEM163 leads to the defective DGs and prolonged bleeding time (17). However, the underlying mechanisms of how TMEM163 is recognized and transported by these HPACs remain undefined. In general, proteins containing LL motifs (XEXXXLL) recognized by adapter proteins traffic among the PM, tubular endosomes, endolysosomal compartments, or the Golgi network *via* adaptor-mediated trafficking (23, 24). Bioinformatic analysis revealed a LL motif at its N-terminus (residues 64–70) of TMEM163. We here demonstrated that this specific LL-based sorting signal in TMEM163 is recognized by either BLOC-1 or AP-3, but not by AP-1, AP-2, or BLOC-2. We characterized that both BLOC-1 and AP-3 participate in TMEM163 trafficking *via* the shared LL sorting motif, revealing an unusual competitive sorting mechanism utilized by different HPACs for LRO biogenesis.

## Results

### Deficiencies in BLOC-1, BLOC-2 and AP complexes (AP-1, -2, -3) reduce TMEM163 protein levels in megakaryocytic cells

Although neuronal and endocrine cell lines (such as PC12, COS-7) are widely used in general secretory vesicle transport studies, given that megakaryocytes act as platelet progenitor cells and are responsible for the production and release of platelets into the peripheral circulation. Therefore, we chose the MEG-01 megakaryocyte line as the model system to study the sorting mechanism of DG precursors by TMEM163, which represents a more physiologically relevant system for investigating platelet DGs.

To dissect the roles of HPACs in TMEM163 sorting, we applied a combined genetic disruption approach in MEG-01 cells. This included knocking out AP-3 (*AP3B1*) in WT and in BLOC-1 (*DTNBP1*) KO cells (*D24*) (17) to obtain a single KO (*C8*) and double KO (DKO, *C4*), as well as knocking down (KD) *AP1M1* (AP-1 μ1 subunit), *AP2M1* (AP-2 μ2 subunit), and *HPS6* (BLOC-2 HPS6 subunit), yielding a set of isogenic cell lines (si-*AP1M1*, si-*AP2M1*, and si-*HPS6*) for comparative analysis (Fig. S1*A*). The *AP3B1* gene, containing eight exons, was targeted at exon seven using a pSpCas9 (BB)-2A-GFP (PX458) plasmid (Fig. S1*B*). Sequencing analysis revealed two successful clones: 1) a MEG-01-derived single KO clone (*C8*) with 1-bp insertion in both *AP3B1* alleles (Fig. S1*C*), and 2) a *DTNBP1*-KO-derived DKO clone (*C4*) exhibiting 2-bp deletion in both *AP3B1* alleles (Fig. S1*D*). These mutations are predicted to cause frameshifts and premature stop codons in exon 8. Western blotting validation confirmed null of *AP3B1* in *C8* and *C4* cell lines (Fig. S1*E*).

Consistent with our previous observations in human and mouse genetic models of BLOC-1, BLOC-2, and AP-3 deficiency (17), TMEM163 protein expression was significantly reduced across all corresponding KO/KD MEG-01 cell lines (Fig. S1, *F* and *G*). Notably, this reduction was also observed in AP-1 (*si-AP1M1*) and AP-2 (*si-AP2M1*) KD models (Fig. S1, *H*, *I*, and *F*). This validates the utility of our cellular models for elucidating TMEM163 sorting mechanisms in megakaryocytic cells.

Furthermore, AP-3 KO (*C8*) and AP-3/BLOC-1 DKO (*C4*) cells were incubated with FluoZin-3 and transmission electron microscopy analysis was performed to evaluate the effects of these KO cells. Similar to the *DTNBP1*-KO (17), we observed a higher intracellular Zn^+^ accumulation in granule-like structures in these KO cells (*C8* and *C4*) compared with the control group (Fig. S2). In addition, a significant increase in the number of enlarged round membranous organelles (similar to early endosomes, with reduced lumen contents) in *C8* and *C4* cells was observed (Fig. S3). These indicate that all these KO cells compromise intracellular organellar Zn^+^ homeostasis in platelets and DG biogenesis.

### TMEM163 undergoes specific degradation *via* the proteasome

**Figure 1:**
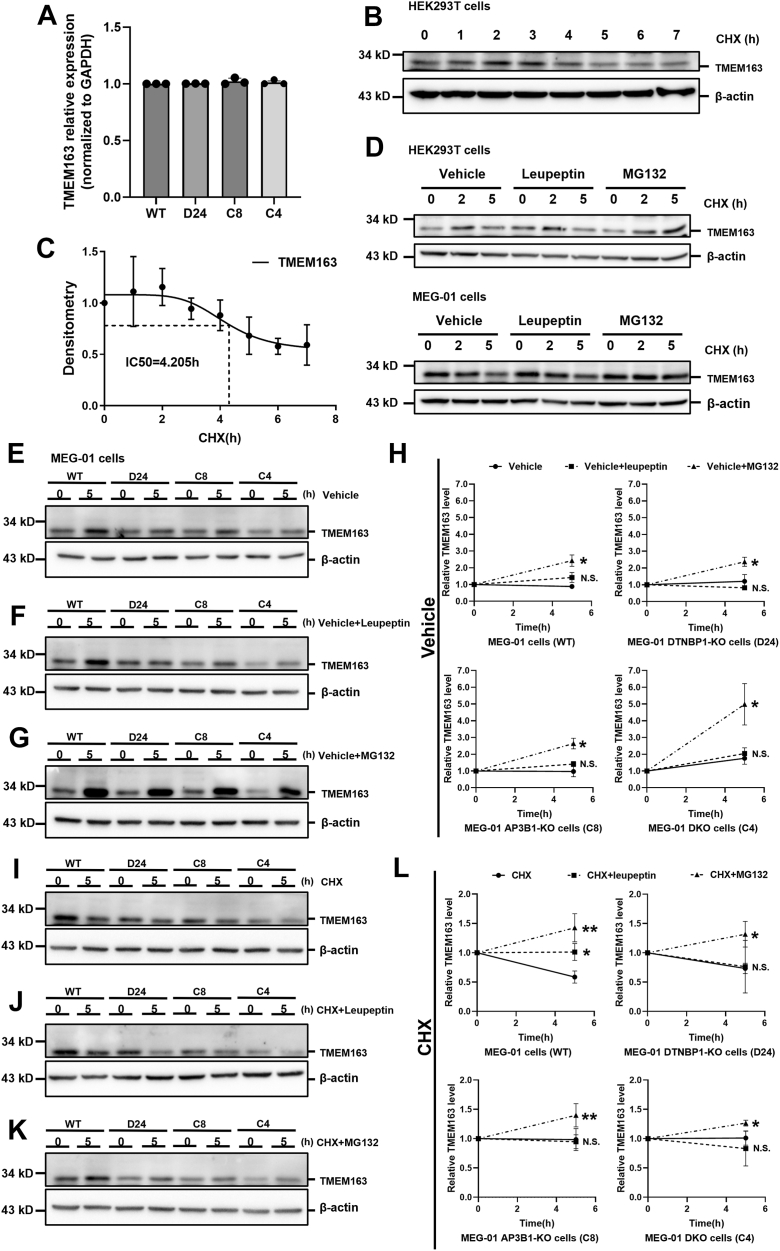
**TMEM163 is selectively degraded by the proteasome.***A*, the expression levels of TMEM163 in WT MEG-01 cells, *DTNBP1-*KO cells, *AP3B1-*KO cells and *DTNBP1/AP3B1* double-KO (DKO) cells were detected by RT-qPCR (n = 3 in each group). One-way ANOVA analysis of variance showed that there was no statistically significant difference among the groups (*p*= 0.4112). Subsequent Tukey’s multiple comparisons test confirmed no significant pairwise differences between any of the groups (all adjusted *p*> 0.05). *B*, HEK293T cells were treated with 20 μM cycloheximide (CHX) and samples were collected every hour for a total of 7 h. The cells underwent Western blotting with endogenous antibodies to TMEM163 to assess the protein levels of TMEM163 and thus detect its half maximal inhibitory concentration (IC50). β-actin serves as a loading control. *C*, the IC50 (=4.205 h) of endogenous degradation of TMEM163 was calculated by GraphPad Prism 9.0. *D*, HEK293T cells and MEG-01 cells were collected at 0, 2, 5 h respectively, and treated with 20 μM CHX and 100 μM leupeptin or MG132. Western blotting was used to analyze the degradation of TMEM163 protein in different cells. *E*–*H*, WT MEG-01 cells, *DTNBP1-*KO cells, *AP3B1-*KO cells, DKO cells treated without (*E*) or with 100 μM leupeptin (*F*) or MG132 (*G*) were collected at 0 h and 5 h. Protein degradation of TMEM163 was evaluated by Western blotting analysis (*H*), represented with solid line for vehicle, dashed line for vehicle and leupeptin, and dash-dot line for vehicle and MG132. β-actin serves as a loading control. *I*–*L*, WT MEG-01 cells, *DTNBP1-*KO cells, *AP3B1-*KO cells, DKO cells treated with 20 μM CHX (*I*) or 20 μM CHX and 100 μM leupeptin (*J*) or 20 μM CHX and 100 μM MG132 (*K*) were collected at 0 h and 5 h. Protein degradation of TMEM163 was evaluated by Western blotting analysis (*L*), represented with solid line for CHX, *dashed line* for CHX and leupeptin, and *dash-dot line* for CHX and MG132. β-actin serves as a loading control. *Bar graphs* represent data from ≥3 independent experiments; each performed with reproducibility confirmed (n = 3). Each *bar* represents the mean ± SD. Paired Student’s *t* test. *Asterisks* above the *bars* indicate significant differences between indicated groups: ∗*p*< 0.05; ∗∗*p*< 0.01; N.S., not significant.

The reduction of TMEM163 levels may be associated with complex mechanisms at various levels, such as transcriptional repression, accelerated protein degradation, and protein instability. To explore these possibilities, we first examined TMEM163 transcription by RT-qPCR analysis of mRNA levels in different stable MEG-01 KO cell lines (*D24, C8* and *C4*). The transcript levels showed unchanged across all cell lines (Fig. 1*A*), excluding transcriptional regulation as a contributing factor.

We then assessed protein stability using cycloheximide (CHX) chase assays in HEK293T cells. Quantification analysis revealed a TMEM163 half-life of 4.205 h (Fig. 1, *B* and *C*), indicating relatively rapid turnover. To identify the degradation pathway, we performed time-course experiments (0/2/5 h) combining CHX with either leupeptin (a lysosome inhibitor) or MG132 (a proteasome inhibitor). MG132 treatment maintained TMEM163 levels in both HEK293T and MEG-01 cells at 5 h post-treatment, whereas leupeptin showed no protective effect compared to DMSO controls (Fig. 1*D*). We expanded these pharmacological interventions to MEG-01 KO lines (*D24*, *C8* and *C4*) under three conditions: 1) vehicle control (DMSO), 2) CHX alone, 3) CHX + inhibitors. Quantification analysis revealed that all cell lines showed progressive TMEM163 accumulation with MG132 treatment, suggesting proteasomal degradation (Fig. 1, *E*–*H*). Following combined CHX treatment, TMEM163 protein levels decreased progressively in both control and leupeptin-treated groups, but were stabilized or increased upon co-treatment of MG132 (Fig. 1, *I*–*L*). In addition, the consistency of these findings was confirmed in *HPS6, AP1M1, AP2M1*-KD cells (Fig. S4, *A*–*D*). Taken together, these results demonstrate that TMEM163 undergoes predominantly proteasomal degradation when the associated trafficking complexes are deficient.

### BLOC-1 and AP-3 adaptor complexes compete for binding to the same dileucine sorting signal of TMEM163

**Figure 2:**
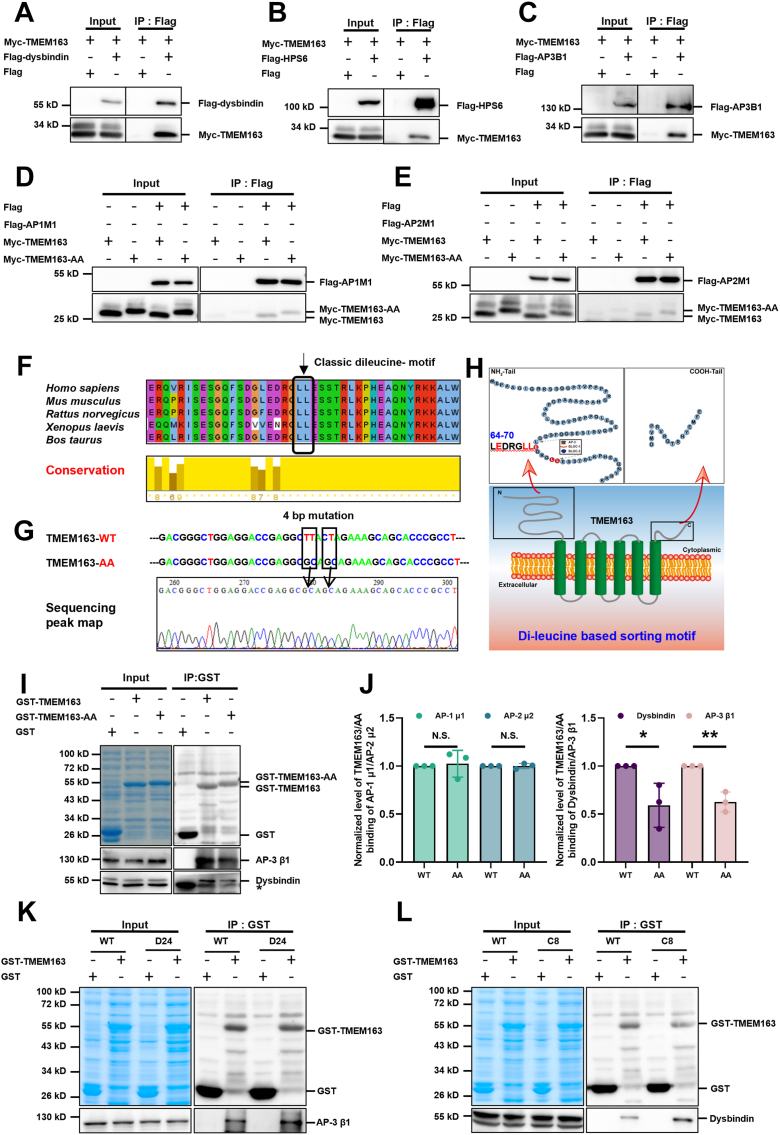
**BLOC-1 and AP-3 adapter complexes compete for a shared dileucine sorting signal in TMEM163.***A*–*C*, interactions of TMEM163 with dysbindin, HPS6, and AP-3 β1. Co-immunoprecipitation assays showed that Myc-TMEM163 co-immunoprecipitated with Flag-dysbindin (*A*), Flag-HPS6 (*B*) and Flag-AP3B1 (*C*). *D* and *E*, interactions of the TMEM163-WT or AA with AP-1 μ1 and AP-2 μ2. Both TMEM163-WT and the TMEM163-AA mutant interacted with AP-1 μ1 and AP-2 μ2 to a comparable extent. *F*, multiple alignments of TMEM163 and homologs. TMEM163 homologs were from *Homo sapiens* (NP_112185.1), *Mus musculus* (NP_082411.1), *Rattus norvegicus* (NP_001104233.2), *Xenopus laevis* (NP_001086283.1), and *Bos taurus* (NP_001094630.1). The *black arrows* indicate the classic dileucine-motif mutation sites. *G*, strategy for point mutation binding motif with AP-3 β1. The genomic changes were marked with black frame. *H*, topology of human TMEM163 and sequence of the cytoplasmic N terminus with putative acidic LL motif. *I*, The GST or GST-TMEM163-WT or GST-TMEM163-AA was mixed with GST-Sep Glutathione MagBeads, incubated with MEG-01 cell lysates, and underwent Western blotting with GST/AP-3 β1/dysbindin antibodies. *A star sign* (∗) indicates a nonspecific band. All the inputs on the *left* correspond to 10% of the entire cell lysate, and the *top* shows a Coomassie-stained gel of identical reactions showing the GST-fusion proteins. *J*, the statistics of AP-1 μ1/AP-2 μ2/dysbindin/AP-3 β1 expression level detected by Western blotting of TMEM163-WT/AA. Input serves as a loading control. Note that the expression of dysbindin/AP-3 β1 showed significant reduction compared to WT, with *p*= 0.0369 and 0.0034, respectively, while the expression of AP-1 μ1/AP-2 μ2 was not significantly different compared to WT, with *p*= 0.7823 and 0.9874 respectively; each bar represents the mean ± SD, n = 3. Unpaired Student’s *t* test. ∗*p*< 0.05; ∗∗*p*< 0.01. *K and L*, GST or human GST-TMEM163 was mixed with GST-Sep Glutathione MagBeads, and incubated with the MEG-01 WT/*D24*/*C8* cell lysates. The bound proteins were separated by SDS polyacrylamide gel electrophoresis and Western blotting was performed using antibodies against GST and AP-3 β1/dysbindin.

Our data have shown that TMEM163 expression is reduced across a variety of cells or tissues with different complex deficiencies (Fig. S1*F*) (17). Based on these findings, we hypothesized that TMEM163 may physically interact with these complexes. Co-immunoprecipitation experiments confirmed that TMEM163 interacts with core subunits of BLOC-1 (dysbindin), BLOC-2 (HPS6), AP-1 (μ1), AP-2 (μ2), and AP-3 (β1) (Fig. 2, *A*–*E*), supporting the notion that TMEM163 functions as a cargo protein transported by these complexes. However, how these complexes are involved in the trafficking of TMEM163 remains elusive.

Given that the HPAC complex typically recognizes cargo proteins *via* conserved sorting signals such as acidic dileucine (LL) motifs ([D/E]XXXL[L/I]) that mediate interactions with heterotetrameric adapter proteins (*e.g.*, AP-1, AP-2 and AP-3), we searched the eukaryotic linear motifs database for similar motifs within TMEM163. This analysis identified a potential di-leucine motif (LEDRGLL) at residues 64 to 70 within its N-terminal domain. A cross-species conservation analysis revealed that Leu69 and Leu70 residues within the identified TMEM163 motif are highly conserved (Fig. 2*F*). We therefore generated a TMEM163 mutant in which both leucines (TMEM163-WT) were substituted with alanines (TMEM163-AA) through site-directed mutagenesis (Fig. 2, *G* and *H*).

To assess whether this motif mediates binding to HPACs, we performed Co-immunoprecipitation experiments and found that both TMEM163-WT and TMEM163-AA mutant interacted with AP-1 μ1 and AP-2 μ2 to a similar extent (Fig. 2, *D* and *E*), indicating that the LEDRGLL motif is not essential for AP-1 or AP-2 binding. In contrast, affinity pull-down assays using recombinant GST-tagged TMEM163 and TMEM163-AA proteins revealed that the AA mutant significantly impaired the interaction with endogenous dysbindin (a BLOC-1 subunit) and AP-3 β1 (Fig. 2, *I* and *J*). These results suggest that the dileucine motif is specifically required for TMEM163 binding to BLOC-1 and AP-3.

We further verified these interactions by incubating with lysates from WT, *AP3B1-KO* (*C8*) and *DTNBP1-KO* (*D24*) MEG-01 cells. Western blotting analysis revealed GST-TMEM163 bond both dysbindin and AP-3 β1 in WT cells. Intriguingly, in the absence of dysbindin (*D24* lysate), the association between TMEM163 and AP-3 β1 was markedly enhanced (Fig. 2*K*). Conversely, the absence of AP-3 β1 (*C8* lysate) enhanced the interaction between TMEM163 and dysbindin (Fig. 2*L*). Together, these data demonstrate that BLOC-1 and AP-3 compete for binding to the same dileucine sorting signal on TMEM163, revealing an unusual and novel mechanism for cargo sorting where two distinct adapter complexes recognize the same motif in a mutually competitive manner.

### AP-3 and BLOC-1 deficiency compromise the targeting of TMEM163 to DG precursors

To further investigate the unusual cargo-sorting mechanism involving TMEM163, we systematically assess its subcellular localization through live-cell imaging in combination with different organelle markers. Based on our previous findings that TMEM163 localizes to DG precursors and early endosomal compartments in MEG-01 cells (17), we performed quantitative colocalization analysis using Pearson’s correlation coefficient (PCC).

**Figure 3:**
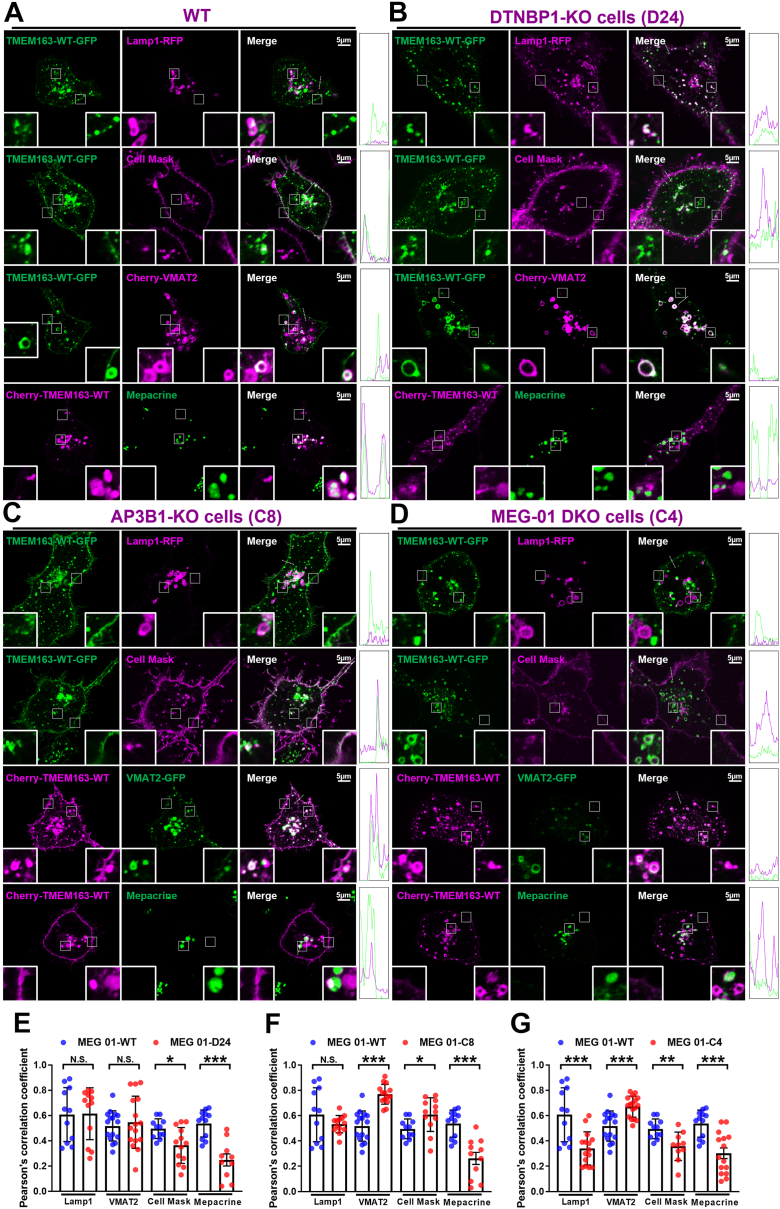
**Live-cell imaging of TMEM163 in dense granule precursors and plasma membrane in AP-3 and BLOC-1 deficient MEG-01 cells.***A*–*D*, WT MEG-01 cells (*A*), *DTNBP1-*KO cells (*B*), *AP3B1-*KO cells (*C*), DKO cells (*D*) were co-transfected with GFP/Cherry-tagged TMEM163, Lamp1-RFP, GFP/Cherry-tagged VMAT2 for 24 h. For cell membrane localization, the cells were transfected with TMEM163-GFP for 24 h, and then treated with Cell Mask plasma membrane staining at 37 °C for 10 min. For dense granule precursor labeling, the cells were transfected with Cherry-TMEM163 for 24 h then treated with 50 μM mepacrine for 30 min at 37 °C. The transfected and stained cells were photographed in living cell dishes. The *white box regions* on the right-bottom represent PM localization, and the *white box regions* on the *left-bottom* represents the intracellular localization which are magnified 4-fold in the insets. The scale bar represents: 5 μm. The 5 μm white *dashed lines* in the merge panel outline regions of PM used for fluorescence intensity quantification across *green* and *magenta* channels. Line scan profiles of the corresponding fluorescence intensities are provided in the *right rectangles*. *E* and *G*, comparison of different KO cell lines with WT in intracellular statistics by colocalization analysis of PCC, respectively. PCC in Lamp1 (WT/*D24*/*C8/C4*, 0.60 ± 0.06/0.61 ± 0.05/0.53 ± 0.01/0.34 ± 0.03); PCC in VMAT2 (WT/*D24*/*C8*/*C4*, 0.51 ± 0.02/0.54 ± 0.05/0.76 ± 0.02/0.67 ± 0.02); PCC in Cell Mask (WT/*D24*/*C8*/*C4*, 0.49 ± 0.02/0.36 ± 0.03/0.60 ± 0.03/0.35 ± 0.03); PCC in mepacrine (WT/*D24*/*C8/C4*, 0.53 ± 0.03/0.24 ± 0.04/0.26 ± 0.04/0.30 ± 0.04). Data represent mean ± SD across 10 to 21 cells for each sample. Unpaired Student’s *t* test. ∗*p*< 0.05; ∗∗*p*< 0.01; ∗∗∗*p*< 0.001; N.S., not significant. PCC, Pearson’s correlation coefficient; PM, plasma membrane.

To evaluate whether TMEM163 is correctly targeted to lysosomes or DGs, we examined the colocalization of TMEM163 with a lysosomal marker (Lamp1-RFP) and DG markers (Cherry-VMAT2 and mepacrine). In *DTNBP1-*KO cells (*D24*), colocalization of TMEM163 with Lamp1 and VMAT2 remained unchanged relative to WT controls. (Fig. 3, *A*, *B*, and *E*). Intriguingly, both AP-3 β1-deficient (*C8* and *C4*) cell lines exhibited enhanced TMEM163-VMAT2 colocalization (Fig. 3, *C*, *D*, *F*, and *G*). Previous studies have demonstrated that mutation of the VMAT2 cytosolic tail sorting signal (mutating EEKMAIL to EEKMAAA) causes mislocalization of the mutant protein to the PM in MEG-01 cells (25). This finding indicates that AP-3 recognizes sorting signals mediating VMAT2 transport to DGs. Consequently, in AP-3 β1-deficient MEG-01 cells (*C8* and *C4*), VMAT2 is likely missorted to the PM or other endocytic organelles. In addition, it is plausible that loss of AP-3 directly impairs the biogenesis of mature DGs or lysosomes. This defect would disrupt the normal subcellular localization of VMAT2 and TMEM163, ultimately leading to an artifact that manifests as an apparent increase in TMEM163-VMAT2 intracellular co-localization. Indeed, we observed reduced colocalization of TMEM163 and mepacrine across all cell lines (*D24, C8*, *C4*), a marker of mature DG (Fig. 3), suggesting the reduced targeting of TMEM163 to DGs.

### AP-3 and BLOC-1 deficiency lead to differentially mislocalized TMEM163 on endo-lysosomal compartments

The PM colocalization patterns differed in different genotypes: *D24* and *C4* showed reduced Cell Mask colocalization, whereas *C8* displayed increased PM accumulation (Fig. 3). This suggests that loss of AP-3 leads to increased PM localization probably *via* recycling endosomes (REs), while loss of BLOC-1 leads to TMEM163 degradation thus exhibiting reduced localization on the PM.

**Figure 4:**
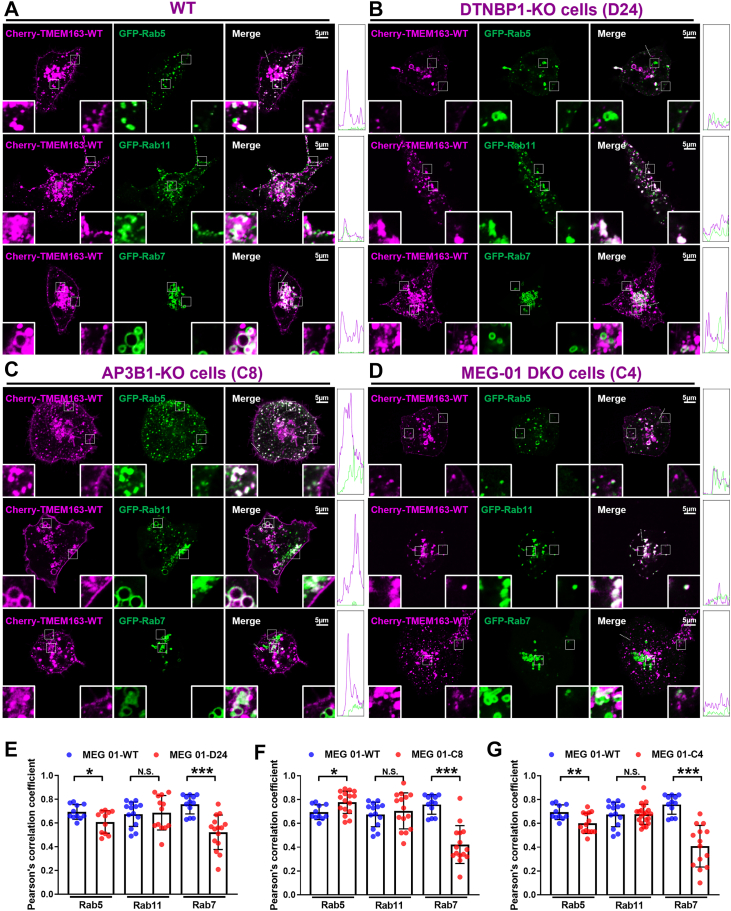
**Live-cell imaging of exogenously expressed TMEM163 with endosomal Rab markers in different MEG-01 cell lines.***A*–*D*, WT MEG-01 cells (*A*), *DTNBP1*-KO cells (*B*), *AP3B1*-KO cells (*C*), DKO cells (*D*) were co-transfected with Cherry-TMEM163 and GFP-Rab5, GFP-Rab11 and GFP-Rab7 for 24 h. The transfected and stained cells were photographed in living cell dishes. The *white box* regions on the *right-bottom* represents PM localization, and the *white box* regions on the *left-bottom* represents the intracellular localization which are magnified 4-fold in the insets. The scale bar represents: 5 μm. The 5 μm *white dashed line*s in the merge panel outline regions of PM used for fluorescence intensity quantification across *green* and *magenta* channels. Line scan profiles of the corresponding fluorescence intensities are provided *right rectangles*. *E* and *G*, comparison of different KO cell lines with WT in intracellular statistics by colocalization analysis of PCC, respectively. PCC in Rab5 (WT/*D24*/*C8*/*C4*, 0.69 ± 0.01/0.61 ± 0.02/0.77 ± 0.02/0.60 ± 0.02); PCC in Rab11 (WT/*D24*/*C8*/*C4*, 0.67 ± 0.02/0.68 ± 0.04/0.70 ± 0.03/0.67 ± 0.01); PCC in Rab7 (WT/*D24*/*C8*/*C4*, 0.75 ± 0.02/0.52 ± 0.03/0.42 ± 0.04/0.40 ± 0.04. PCC ≥ 0.4 represents colocalization. Data represent mean ± SD across 10 to 21 cells for each sample. Unpaired Student’s *t* test. ∗*p*< 0.05; ∗∗*p*< 0.01; ∗∗∗*p*< 0.001; N.S., not significant. PCC, Pearson’s correlation coefficient; PM, plasma membrane

To further reconcile the colocalization profiling in the endocytic pathway, we observed a marked decrease in late endosome (LE, GFP-Rab7) whereas the colocalization with RE (GFP-Rab11) was unaltered across all KO lines (*D24, C8, C4*). The association with early endosome (EE, GFP-Rab5) was slightly decreased in BLOC-1 deficient cells (*D24* and *C4*) but increased in AP-3 deficient cells (*C8*) (Fig. 4), which is similar to ATP8A1 (4). A possible explanation is that TMEM163 fails to interact with AP-3 in *AP3B1*-KO cells, resulting in the accumulation in EE which may be mis-sorted to the PM. However, in the BLOC-1 KO and double AP-3/BLOC-1 KO cells, we observed reduced EE and LE colocalization due to accelerated degradation.

Similar to BLOC-1 KO cells, HPS6 knockdown also led to reduced TMEM163 colocalization with GFP-Rab7, without affecting its colocalization with Lamp1-RFP or Cherry-VMAT2-positive compartments (Fig. S5), suggesting that BLOC-1 and BLOC-2 may act in the same trafficking route.

**Figure 5:**
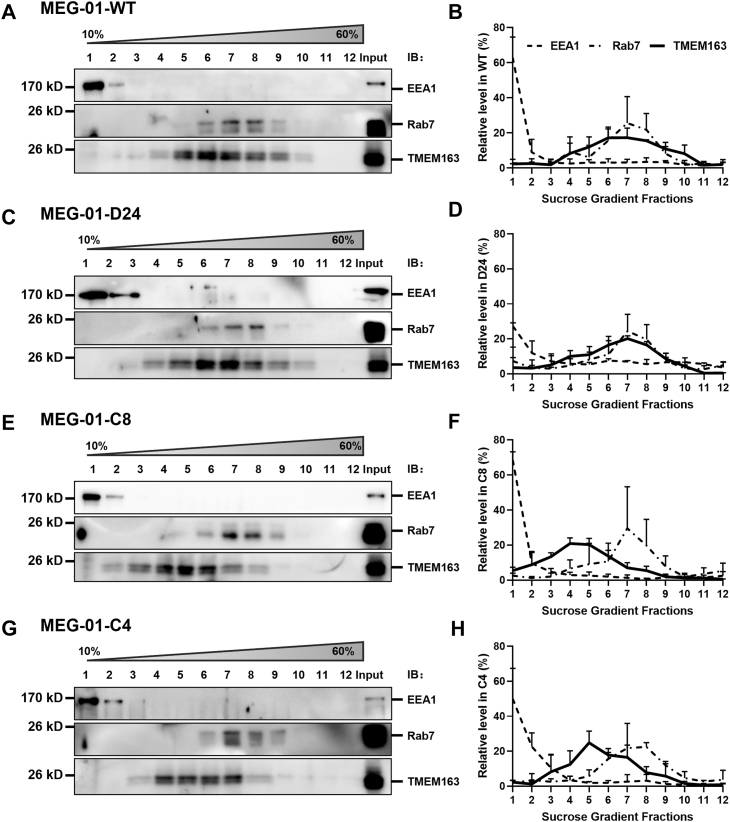
**TMEM163 co-fractionates with endosomes in different cell lines.***A*, *C*, *E* and *G*, immunoblotting analysis of fractions obtained from MEG-01 and other KO cell lines. Post-nuclear supernatants were subjected to subcellular fractionation with a 10 to 60% sucrose gradient. EEA1, EE marker; Rab7, LE marker. *B, D*, *F* and *H*, the distribution curves were plotted by the percentage of protein expression in each density gradient to the total protein level. In both WT and *DTNBP1*-KO cells (*D24*), TMEM163 distributed across intermediate-density fractions (F4-F10), with no significant differences observed (*e.g., D24* in F4 vs WT, *p*= 0.6744; *D24* in F7 vs WT, *p*= 0.4289). In contrast, *AP3B1-*KO cells (*C8*) exhibited a significant leftward shift towards lower density fractions (F2-F8; *e.g. C8* in F4 vs WT, *p*= 0.0312; *C8* in F7 vs WT, *p*= 0.0462). DKO cells (*C4*) also showed a density reduction (F3-F7; *e.g., C4* in F5 vs WT, *p*= 0.0661; *C4* in F7 vs WT, *p*= 0.8889). Each *bar* represents the percentage (mean ± SD) for control from three independent experiments. IB, immunoblotting. EEA1 in *dashed line*, Rab7 in *dash-dot line*, TMEM163 in *solid line*.

To further validate the observations of endosomal colocalization, we performed sucrose density gradient centrifugation to assess membrane associations. Compartment-specific markers were used including EEA1 (EE) and Rab7 (LE). Normalized distribution profiles were generated through densitometric analysis. The results showed that the distribution patterns of EE and LE markers were similar across all genotypes (EEA1 at F1-F2; Rab7 at F5-F9). In *DTNBP1-*KO (*D24*) cells, TMEM163 distributed across intermediate-density fractions (F4-F10) similar to WT (Fig. 5, *A*–*D*). In contrast, *AP3B1-*KO *(C8)* cells exhibited a significant leftward shift towards lower density fractions (F2-F8) (Fig. 5, *E* and *F*). Likewise, DKO cells (*C4*) showed a significant left-shifting to low density reduction (F3-F7) (Fig. 5, *G* and *H*). Collectively, these data indicate that in WT cells, TMEM163 primarily resides in Rab7-positive compartments. AP-3 deficiency disrupts anterograde trafficking from EE to downstream LE, leading to the abnormal accumulation of TMEM163 in low-density membrane fractions containing EEs. However, the leftover TMEM163 in *DTNBP1*-KO cells was in LEs. This agrees with the endosomal colocalization profiles (Fig. 4).

### The di-leucine motif in TMEM163 mediates AP3-dependent intracellular trafficking to prevent its mislocalization to the PM

**Figure 6:**
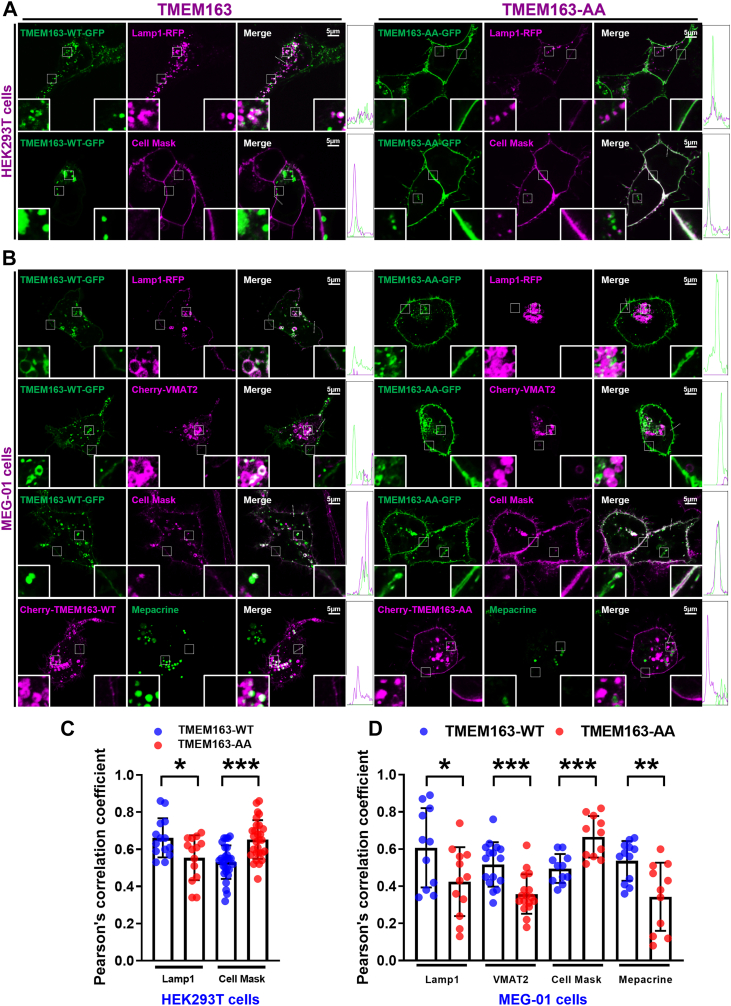
**Live-cell imaging of exogenously expressed GFP/Cherry-tagged TMEM163-WT/AA in HEK293T cells and MEG-01 cells.***A*, HEK293T cells were cotransfected with TMEM163-WT-GFP or TMEM163-AA-GFP and Lamp1-RFP for 24 h, respectively, then treated with Cell Mask PM staining at 37 °C for 10 min for cell membrane localization. The transfected and stained cells were photographed in living cell dishes. *B*, MEG-01 cells were co-transfected with TMEM163-WT-GFP or TMEM163-AA-GFP and Lamp1-RFP or Cherry-VMAT2 for 24 h, and then treated with Cell Mask PM staining at 37 °C for 10 min for cell membrane localization. The cells were transfected with Cherry-TMEM163-WT or Cherry-TMEM163-AA for 24 h and then treated with 50 μM mepacrine for 30 min at 37 °C. The transfected and stained cells were photographed in living cell dishes. The *white box* regions on the *right-bottom* represent PM localization, and the *white box* regions on the *left-bottom* represents the intracellular localization which are magnified 4-fold in the insets. The scale bar represents: 5 μm. The 5 μm white dashed lines in the merge panel outline regions of PM used for fluorescence intensity quantification across *green* and *magenta* channels. Line scan profiles of the corresponding fluorescence intensities are provided right rectangles. *C* and *D*, comparison of different cell lines with WT in intracellular statistics by colocalization analysis of PCC, respectively. HEK293T cells (*C*): PCC in Lamp1 (TMEM163 vs TMEM163-AA, 0.66 ± 0.02 vs 0.55 ± 0.03); PCC in Cell Mask (TMEM163 vs TMEM163-AA, 0.53 ± 0.01 vs 0.65 ± 0.01). MEG-01 cells (*D*): PCC in Lamp1 (TMEM163 vs TMEM163-AA, 0.60 ± 0.06 vs 0.42 ± 0.05); PCC in VMAT2 (TMEM163 vs TMEM163-AA, 0.51 ± 0.02 vs 0.35 ± 0.02); PCC in Cell Mask (TMEM163 vs TMEM163-AA, 0.49 ± 0.02 vs 0.66 ± 0.03). PCC in mepacrine (TMEM163 vs TMEM163-AA, 0.53 ± 0.03 vs 0.34 ± 0.05). PCC ≥ 0.4 represents colocalization. Data represent mean ± SD across 11 to 28 cells for each sample. Unpaired Student’s *t* test. ∗*p*< 0.05; ∗∗∗*p*< 0.001; N.S., not significant. PCC, Pearson’s correlation coefficient; PM, plasma membrane

To further substantiate these findings, we investigated the subcellular localization of TMEM163-AA mutant in both HEK293T and MEG-01 cells by live-cell imaging and quantified the colocalization using PCC. In HEK293T cells, TMEM163-AA exhibited significantly reduced colocalization with lysosomes (Lamp1-RFP) and LE (GFP-Rab7) markers, while showing increased PM association (Cell Mask). In contract, no significant differences were observed in colocalization with EE (GFP-Rab5) or RE (GFP-Rab11) markers (Figs. 6, *A* and *C* and S6, *A* and *C*). Parallel experiments in MEG-01 cells corroborated these findings, showing decreased colocalization with lysosomal (Lamp1-RFP), DG (Cherry-VMAT2, mepacrine), and LE (GFP-Rab7), along with enhanced PM localization (Figs. 6, *B* and *D* and S6, *B* and *D*). These results demonstrated that TMEM163-AA had fewer intracellular localization but with increased PM localization compared to WT, which resembles those observed in *AP3B1-*KO cells (*C8*) (Fig. 3, *C* and *F*).

Given that AP-1 typically regulates the sorting of cargo proteins from the trans-Golgi network, while AP-2 mediates the internalization of cargos from the PM into endosomes, we sought to investigate the roles of these APs in TMEM163 trafficking. As expected, knockdown of AP-1 μ1 reduced the fraction of TMEM163 with the PM, whereas knockdown of AP-2 μ2 increased its PM localization (Fig. S7). Although both AP-1 and AP-2 were found to interact with TMEM163, the LL motif was not essential for binding to either AP-1 or AP-2 (Fig. 2, *D*, *E*, and *J*). The sorting motifs of AP-1 and AP-2 are to be identified in future as this study is focusing on the trafficking of TMEM163 mediated by HPACs.

## Discussion

Our study provides mechanistic insights into the sorting of TMEM163 to LROs such as platelet DGs, identifying it as a cargo protein reliant on the synergistic coordination of the AP-3 complex and the BLOC-1 complex, mediated through a conserved N-terminal cytosolic dileucine motif (LEDRGLL; 64–70 AA), which is the critical sorting signal recognized by both AP-3 and BLOC-1. This aligns with canonical adapter-cargo recognition principles wherein acidic dileucine motifs ([D/E]XXXL[L/I]) serve as universal sorting signals for endolysosomal trafficking (10).

**Figure 7:**
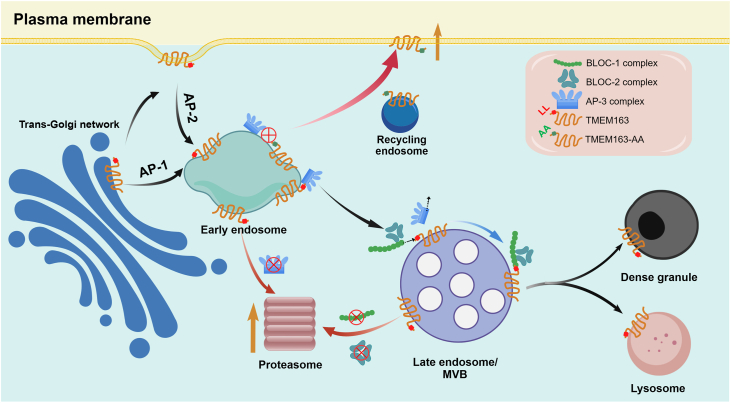
**A model depicting the regulatory role of HPAC complexes in TMEM163 trafficking.** AP-1 complex mediates transport of TMEM163 from the trans-Golgi network, while AP-2 complex promotes its internalization from the PM into the endo-lysosomal compartments. Upon reaching the early endosomes, the dileucine (LL) sorting motif of TMEM163 is recognized by AP-3 complex to direct it into the late endosomes where BLOC-1 complex competes for the binding of the LL motif, thereafter targeting to the dense granules or lysosomes. When the LL motif is mutated to AA, TMEM163 is missorted to the PM. Similarly, in AP-3 deficient cells, TMEM163 is misrouted to the PM possible *via* the recycling endosomes, but mostly subjects to accelerated degradation in the proteasomes. In contrast, in BLOC-1/2 deficient cells, the endo-lysosomal trafficking of TMEM163 is hampered at the endosomal compartments where it triggers proteasomal degradation. PM, plasma membrane

Both AP-3 and BLOC-1 complexes bind directly to TMEM163 *via* the same LL motif. Intriguingly, loss of one complex (BLOC-1 deficiency in *D24* cells) significantly enhanced TMEM163 binding to the other complex (AP-3 deficiency in *C8* cells), and *vice versa*. This suggests potential competition between AP-3 and BLOC-1 for the LL signal during trafficking. The mislocalization effects, notably the paradoxical PM and EE accumulations in *AP3B1-*KO (*C8*) *versus DTNBP1-*KO (*D24*) and DKO (*C4*) cells, lead us to speculate that BLOC-1 and AP-3 sequentially mediate TMEM163 for DG targeting. Our data suggest that AP-3 directs TMEM163 from EE to LE. BLOC-1 (thereafter BLOC-2) directs TMEM163 from LE to DG by competitively binding to the same LL sorting motif. When this DG targeting route is disrupted, most of TMEM163 is subjected to proteasomal degradation from EE or LE. However, in AP-3 deficiency or in the TMEM163-AA mutant, TMEM163 is accumulated in the EE, which is mis-targeted to the PM (Fig. 7).

In the trafficking of other LRO cargoes such as tyrosinase (TYR), OCA2 and TYRP1 to the melanosomes, AP-3 and BLOC-1 act differently or synergistically (2, 3). TYR is sorted into AP-1 or AP-3 endosomal buds through a LL motif and then transported into melanosomes *via* the BLOC-1-independent pathway. OCA2 is sorted into AP-3 buds through a LL motif and thereafter is directed by BLOC-1 into mature melanosomes. TYRP1 is sorted into AP-1 buds and then to mature melanosomes *via* the BLOC-1-dependent pathway, but independent of AP-3 (10, 11). Moreover, through si-*HPS6*, we observed that the localization of TMEM163 in the endosomes was similar to that in *DTNBP1-*KO *(D24)* cells. This is consistent with the previous findings that BLOC-1 can interact with BLOC-2 and promote the transport of TYRP1 through a mechanism apparently independent of the function of AP-3 (26), suggesting that BLOC-1 and BLOC-2 may play synergistic roles in the transport of TMEM63 along the same pathway. Thus, TMEM163 can be sorted into LROs in a manner that differs from TYR (AP-3 dependent but BLOC-1 independent), TYRP1 (BLOC-1 dependent but AP-3 independent), but similar to OCA2 (both AP-3 and BLOC-1 sequentially dependent).

Although OCA2, TYR and other membrane proteins can be recognized by AP-3 through LL motifs (4, 6, 7, 11, 12), their endo-lysosomal trafficking machinery utilized may be different. Whether there would be a generalized cargo sorting mechanism applied to HPACs in endo-lysosomal trafficking requires further exploration. Beyond the LL recognition motif, some cargo proteins can be recognized by the HPACs through the tyrosine-based signals motif (YXXØ) (10), in which different mechanism may be applied. We here demonstrated a unique mechanism for TMEM163 sorting in which TMEM163 is firstly mediated by AP-3, then is handed over to BLOC-1/-2 through a competitive binding manner.

The altered trafficking of TMEM163 in endosomal compartments likely exposes hydrophobic domains or misfolded regions to ubiquitin ligases, significantly enhanced proteasomal degradation as observed across all AP-3-, BLOC-1- and BLOC-2-deficient cell lines, providing an explanation for its reduction in HPAC-deficient cells, mice and human patients (17). This represents a unique quality control pathway for specific LRO cargoes, distinct from lysosomal protein trafficking mediated by AP-3 (27).

It seems that the mechanisms of cargo trafficking mediated by the HPACs are differently regulated dependent on the cargoes, cell types or targeted LROs. Our findings reveal an unusual sorting mechanism utilized by AP-3 and BLOC-1 through competitively binding to the same LL sorting signal, which may be applied to other HPAC cargoes. Moreover, these findings are important for the understanding of the underlying mechanism of DG defects in patients with *TMEM163* mutation or some subtypes of HPS involving deficiencies of AP-3, BLOC-1 or BLOC-2 complex.

## Experimental procedures

### Antibodies and reagents

The experiments were conducted using the following antibodies and reagents. The polyclonal (pAb) rabbit anti-mouse dysbindin/HPS7/DTNBP1 antiserum for immunoblotting was generated in-house. The following commercial antibodies were used: rabbit anti-TMEM163 pAb (HPA007224, Sigma-Aldrich) and rabbit anti-TMEM163 pAb (228002/228003, Synaptic Systems). Additional antibodies included: rabbit anti-Myc pAb (C3956, Sigma-Aldrich), mouse anti-β-actin mAb (A5441, Sigma-Aldrich), mouse anti-Flag mAb (F7425, Sigma-Aldrich), rabbit anti-Rab7 mAb (9367, Cell Signaling Technology), rabbit anti-AP3B1 pAb (13384-1-AP, Proteintech), mouse anti-EEA1 mAb (610457, BD Pharmingen), rabbit anti-GST pAb (Bs-2122R, Bioss) and HRP-conjugated mouse anti-rabbit IgG (BE0107, Easybio). Other reagents used in this study are: TPA (12-O-tetradecanoylphorbol-13-acetate; #4174, Cell Signaling Technology), mepacrine (Q2876, Sigma-Aldrich), Cell Mask Deep Red (C10046, Thermo Fisher Scientific), KOD One PCR Master Mix (KMM-101, TOYOBO), Anti-FLAG agarose beads (M8823, Sigma-Aldrich), GST-Sep lutathione MagBeads (20562ES, Yeasen), JetOptimus transfection reagent (10100006, Polyplus), Lipofectamine 2000 (11668-019, Invitrogen), HyClone RPMI 1640 media (SH30809.01, HyClone), DMEM High Glucose Hepes (12430054, Gibco), FBS (10099-141, Gibco), Protease inhibitor cocktail (P8340, Sigma-Aldrich), RNeasy Mini Kit (74104, QIAGEN), iScript cDNA Synthesis Kit (1708891, BIO-RAD), SuperReal Fluorescence Quantitative Premix Plus (SYBR Green) Kit (FP205-02, TIANGEN), RNAiMAX (13778150, Thermo Fisher Scientific) and FluoZin-3 (F24195, Life Technologies).

### Site-directed mutagenesis

TMEM163 dileucine motifs were predicted using the Eukaryotic Linear Motif database (http://elm.eu.org/). The Leu69/Leu70-to- Ala mutant (TMEM163-AA) was generated *via* PCR mutagenesis (KOD One PCR Master Mix, TOYOBO, KMM-101) and validated by Sanger sequencing. Plasmid expression was confirmed by western blotting in HEK293T and MEG-01 cells.

### Reverse transcription quantitative polymerase chain reaction (RT-qPCR) analysis

Total RNA was extracted from MEG-01 cells using the RNeasy Mini Kit (QIAGEN, # 74104). RNA quality was verified by measuring the OD260/280 ratio with a spectrophotometer, ensuring the ratio was between 1.8 and 2.0. cDNA was synthesized from 1 μg of total RNA using the iScript cDNA Synthesis Kit (BIO-RAD, # 1708891). RT-qPCR experiments were conducted using the SuperReal Fluorescence Quantitative Premix Plus (SYBR Green) Kit (TIANGEN, # FP205-02) with an appropriate amount of cDNA as the template. Melting curve analysis was performed to confirm specific amplification. TMEM163 expression was calculated using the 2^−ΔΔCt^ method, normalized to *GAPDH* as the reference gene, and compared with the control group. All samples were run in triplicate.

### Cell culture and transfection

MEG-01 cells were maintained in RPMI-1640 with 10% fetal bovine serum (FBS) (Gibco, #10099-141) and 1% penicillin/streptomycin at 37 °C and 5% CO_2_, passaged every 2 to 3 days, and transfected with JetOptimus during TPA-induce differentiation. HEK293T cells were cultured in DMEM medium supplemented with 10% FBS and 1% penicillin/streptomycin at 37 °C and 5% CO_2_, passaged every 2 to 3 days, and transfected with Lipofectamine 2000 (Invitrogen, #11668-019) at 80 to 90% confluence.

### Generation of MEG-01 KO cell lines

sgRNA targeting human *AP3B1* (Gene ID: 8546) was designed using the CRISPR Design Tool (http://tools.genome-engineering.org) (28). The sgRNA (ACATGCTAACTCGATATGCT, exon 7) was cloned into PX458 and transfected into MEG-01 cells using JetOptimus. Single cells were sorted by Fluorescence-Activated Cell Sorting, expanded, and validated by genomic PCR, Sanger sequencing and immunoblotting.

### siRNA treatment

The sense strands of the following siRNAs were synthesized by GenePharma, with the selection of the following mRNA sequences as targets for knockdown: CCCGATCAGTGTCAAGTTCGA (μ subunit of AP-1) (29), AGUGGAUGCCUUUCGGGUCAUU (μ subunit of AP-2) (GenePharma) and GCUGGGAGGAAGGUCCUATT (HPS6 subunit of BLOC-2) (30). A total of 20 nM of siRNAs were introduced into MEG-01 or HEK293T cells utilizing Lipofectamine RNAiMAX transfection reagent, following the manufacturer's protocol. After transfection, the cells were incubated for 48 h for further experimental procedures.

#### Immunoblotting (Western blotting)

Cells were lysed in lysis buffer (50 mM Tris· HCl, pH 7.4, 150 mM NaCl, 0.1% SDS, 1% Triton X-100, 1% sodium deoxycholate) with protease inhibitor cocktail (Sigma-Aldrich, P8340), boiled with loading sample buffer and then subjected to 10 to 15% SDS polyacrylamide gel electrophoresis (SDS-PAGE). Immunoblotting procedures and quantitative analysis of protein bands were described previously (31, 32).

#### Co-immunoprecipitation and immunoprecipitation assays

HEK293T cells were seeded in 6-well plates and incubated at 37 °C with 5% CO_2_ until they reached 70 to 80% confluency (typically 16–24 h). Subsequently, 4 μg of DNA (including 2 μg each of the following co-transfection plasmids: Flag-empty vector, Flag-dysbindin, Flag-HPS6, Flag-AP3B1, and Myc-TMEM163) was transfected using JetOptimus (Polyplus, USA). After 24 h, the cells were lysed in lysis buffer with protease inhibitor cocktail for 30 min at 4 °C, and the supernatant containing the total protein lysate was collected. 10% of the lysate was used as input, while 90% was incubated with anti-FLAG agarose beads at 4 °C for 6 h. The beads were then washed six times to remove non-specific binding, and bound proteins were eluted by boiling in 2× SDS loading buffer for 10 min. Finally, the eluted proteins and input samples were separated by 10% SDS-PAGE.

### GST pull-down assays

GST-empty vector and GST-TMEM163 fusion constructs were expressed in *E. coli* Rosetta (DE3). When the A_600_ of the bacterial culture reached 0.8, protein expression was induced by adding 0.6 mM IPTG, followed by incubation at 16 °C for 16 h. The GST fusion proteins were then incubated with GST-Sep Glutathione MagBeads (Yeasen, 20562ES) at 4 °C for 2 h. Subsequently, the beads were washed alternately twice with 1 mL PBS and twice with 1 mL high-salt buffer (20 mM imidazole, 1 M NaCl, 1 mM EDTA, 1 mM DTT).

MEG-01 cells, *DTNBP1-*KO MEG-01 cells, and *AP3B1-*KO MEG-01 cells were collected, and lysed in buffer containing protease inhibitor cocktail at 4 °C for 30 min, and centrifuged to obtain total cell lysates. 10% of each cell lysate was used as input, while 90% was incubated with the washed MagBeads at 4 °C for 2 h. The beads were then washed six times to remove non-specific binding, and bound proteins were eluted by boiling in 2× SDS loading buffer for 10 min. Finally, the eluted proteins and corresponding input samples were separated by 10% SDS-PAGE.

### Sucrose density gradient fractionation

MEG-01 cells were homogenized in HB buffer (0.32 M sucrose, 20 mM Hepes, pH 7.4) with protease inhibitor cocktail, and centrifuged for 15 min at 800g. Post-nuclear supernatants were layered onto a linear 10 to 60% sucrose gradients and ultracentrifuged at 113 000g for 16 h in a SW41 rotor at 4 °C. Twelve equal fractions (F1 representing the least density to F12 the most density) were collected. Proteins were precipitated using trichloroacetic acid-deoxycholic acid sodium-acetone and analyzed by immunoblotting. Detail procedures were described previously (17).

### Immunofluorescence and confocal imaging

MEG-01 cells were cultured on Nunc Lab-Tek chambers (Thermo Fisher Scientific) and transfected with the indicated plasmids for 4 h using JetOptimus following the manufacturer's instructions and treated with 200 nM TPA for 12 to 16 h. HEK293T cells were cultured on 35 mm glass-bottom dishes and transfected with the indicated plasmids for 4 h using Lipofectamine 2000 (Invitrogen, 11668-019) following the manufacturer's instructions for 12 to 16 h. All images were acquired on an LSM 880 confocal Airscan microscope (Zeiss). PCCs were calculated using NIH ImageJ (Pearson’s coefficient ≥ 0.4 defined colocalization).

### Statistical analysis

Images were processed in ZEN 3.7 (Zeiss) and analyzed with Fiji (Coloc2 plugin). Data from ≥ 3 independent experiments were presented as mean ± SD. All the quantitative results were plotted by one-way ANOVA with Tukey’s post-test, except where specified otherwise such as Student’s *t* test. ∗*p*< 0.05; ∗∗*p*< 0.01; ∗∗∗*p*< 0.001; N.S., not significant. All graphs were drawn in GraphPad Prism 9.5.1 (La Jolla, CA, USA; https://www.graphpad.com). This graph in Figure 7 is generated through BioGDP.com (33).

## Data availability

All materials, including the raw data for analysis and figure generation, plasmids, and the cell lines generated in this study, can be obtained from the corresponding authors upon reasonable request.

## Supporting information

This article contains supporting information materials in Tables S1–S7, and supporting data in Figs. S1–S7.

## Conflict of interest

The authors declare that they have no conflicts of interest with the contents of this article.
