# Supplementary material for: A dileucine motif in TMEM163 is essential for its binding with both AP-3 and BLOC-1 complex
Source: J Biol Chem. 2026 Apr 14;302(6):111451. doi: 10.1016/j.jbc.2026.111451 (PMC13195334; doi:10.1016/j.jbc.2026.111451)
Supplement: Supporting information [file mmc1.docx]

**A dileucine motif in TMEM163 is essential for its binding with both AP-3 and BLOC-1 complex**

Zhuang Qi ^1^, Yefeng Yuan ^1^ *, and Wei Li ^1, 2, 3^ *

**Supporting information materials**

**Table S1. Plasmids information**

| **Plasmid Name** | **Description** | **Source/Construction** |
| --- | --- | --- |
| pSpCas9(BB)-2A-GFP (PX458) | Plasmid backbone | A gift from Prof. Runlin Z. Ma’s lab (IGDB) |
| pmCherry-C1-TMEM163 | Cherry-tagged full-length TMEM163 | Our Lab |
| pmCherry-C1-TMEM163-AA | pmCherry-C1-TMEM163 with motif mutated | Site-directed mutagenesis |
| pmCherry-C1-VMAT2 | Cherry-tagged full-length VMAT2 | Our Lab |
| pCMV-Myc-TMEM163 | Myc-tagged full-length TMEM163 | Our Lab |
| pCMV-Myc-TMEM163-AA | pCMV-Myc-TMEM163 with motif mutated | Site-directed mutagenesis |
| Flag-Dysbindin | Flag-tagged full-length Dysbindin | Our Lab |
| Flag-AP1M1 | Expression vector for Flag-tagged μ1 subunit (AP1M1) of AP-1 | Cloned in-house from MEG-01 cDNA |
| Flag-AP2M1 | Expression vector for Flag-tagged μ2 subunit (AP2M1) of AP-2 | Cloned in-house from MEG-01 cDNA |
| Flag-AP3B1 | Expression vector for Flag-tagged β3A subunit (AP3B1) of AP-3 | Cloned in-house from MEG-01 cDNA |
| Flag-HPS6 | Expression vector for Flag-tagged HPS6 subunit of BLOC-2 | Cloned in-house from MEG-01 cDNA |
| pEGFP-N2-TMEM163 | GFP-tagged full-length TMEM163 | Our Lab |
| pEGFP-N2-TMEM163-AA | pEGFP-N2-TMEM163 with motif mutated | Site-directed mutagenesis |
| pEGFP-N2-VMAT2 | GFP-tagged full-length VMAT2 | Our Lab |
| pEGFP-C2-RAB5 | GFP-tagged full-length RAB5 | Our Lab |
| pEGFP-C2-RAB7 | GFP-tagged full-length RAB7 | Our Lab |
| pEGFP-C2-RAB11 | GFP-tagged full-length RAB11 | Our Lab |
| pGEX-GST-TMEM163 | GST-tagged full-length TMEM163 | Our Lab |
| pGEX-GST-TMEM163-AA | pGEX-GST-TMEM163 with motif mutated | Site-directed mutagenesis |
| pCMV-RFP-Lamp1 | RFP -tagged full-length Lamp1 | Our Lab |

**Table S2. Antibodies information**

| **Antibody** | **Host Species** | **Source** |
| --- | --- | --- |
| anti-dysbindin/HPS7/DTNBP1 | Rabbit Polyclonal | in-house |
| anti-TMEM163 | Rabbit Polyclonal | Sigma-Aldrich (HPA007224) |
| anti-TMEM163 | Rabbit Polyclonal | Synaptic Systems (228002/228003) |
| anti-Myc | Rabbit Polyclonal | Sigma-Aldrich (C3956) |
| anti-β-actin | Mouse Monoclonal | Sigma-Aldrich (A5441) |
| anti-Flag | Mouse Monoclonal | Sigma-Aldrich (F7425) |
| anti-Rab7 | Rabbit Monoclonal | Cell Signaling Technology (9367) |
| anti-AP3B1 | Rabbit Polyclonal | Proteintech (13384-1-AP) |
| anti-EEA1 | Mouse Monoclonal | BD Pharmingen (610457) |
| anti-GST | Rabbit Polyclonal | Bioss (Bs-2122R) |
| HRP-conjugated mouse anti-rabbit IgG | Rabbit Polyclonal | Easybio (BE0107) |

**Table S3. Cell lines information**

| **Cell line** | **Species** | **Source** |
| --- | --- | --- |
| HEK293T | Human | ATCC (CRL-3216) |
| MEG-01 | Human | ATCC (CRL-2021) |
| MEG-01 AP3B1-KO cells | Human | This paper |
| MEG-01 DTNBP1-KO cells | Human | This paper |
| MEG-01 DTNBP1/AP3B1-DKO cells | Human | This paper |

All cells were maintained at 37°C in a 5% CO₂ humidified incubator.

**Table S4. Reagents information**

| **Reagent** | **Source** | **Purpose** |
| --- | --- | --- |
| KOD One PCR Master Mix | TOYOBO (KMM-101) | Construction of plasmid mutations |
| Anti-FLAG agarose beads | Sigma-Aldrich (M8823) | Co-IP |
| Lipofectamine RNAiMAX | Thermo Fisher (13778150) | Transfection |
| TPA | Cell Signaling Technology (4174) | Differentiation induction |
| Mepacrine | Sigma-Aldrich (Q2876) | Dense granule marker |
| CellMask Deep Red | Thermo Fisher (C10046) | Plasma membrane labeling |
| GST-Sep Glutathione MagBeads | Yeasen (20562ES) | GST-pulldown |
| JetOptimus transfection reagent | Polyplus (10100006) | Transfection |
| Lipofectamine 2000 | Invitrogen (11668-019) | Transfection |
| HyClone RPMI 1640 media | HyClone (SH30809.01) | Cultivate MEG-01 cells |
| DMEM High Glucose HEPES | Gibco (12430054) | Cultivate HEK293T cells |
| FBS | Gibco (10099-141) | Cell culture nutrient supplement |
| Protease inhibitor cocktail | Sigma-Aldrich (P8340) | Protease inhibition |
| RNeasy Mini Kit | QIAGEN (74104) | RNA extraction |
| iScript cDNA Synthesis Kit | BIO-RAD (1708891) | Reverse Transcription |
| SuperReal Fluorescence Quantitative Premix Plus (SYBR Green) Kit | TIANGEN (FP205-02) | qRT-PCR |
| FluoZin-3 | Life Technologies ( F24195) | Detection of Zn^+^ |

**Table S5. Software and computational tools**

| **Tool** | **Source** | **Purpose** |
| --- | --- | --- |
| Eukaryotic Linear Motif | http://elm.eu.org/ | Prediction of protein sorting motifs |
| Clustal Omega | https://www.ebi.ac.uk/Tools/msa/clustalo/ | Multiple sequence alignment of protein homologs |
| Image J | NIH | Image analysis |
| GraphPad Prism 8.0 | GraphPad Software | Statistical analysis and graphing |
| ZEISS LSM 880 | ZEIS | Confocal laser scanning microscopy |
| BioGDP | BioGDP | Draw the model |
| BD FACSAria Fusion cell sorter | BD | Sort single cells |

**Table S6. siRNA primer sequences information**

| **siRNA** | **Sequences (5’→3’)** |  |
| --- | --- | --- |
| μ subunit of AP-1 | Forward 5’- CCCGATCAGTGTCAAGTTCGA -3’ | |
| μ subunit of AP-2 | Forward 5’- AGUGGAUGCCUUUCGGGUCAUU -3’ | |
| HPS6 subunit of BLOC-2 | Forward 5’- GCUGGGAGGAAGGUCCUATT -3’ | |

**Table S7. qRT-PCR primer sequences information**

|  | **Sequences (5’→3’)** |  |
| --- | --- | --- |
| TMEM163 | Forward 5’- ATTGTCCTGTGGCGTTACAGC-3’  Reverse 5’- AGCCTAGTTGAGAGGTCATGG-3’ | |
| GAPDH | Forward 5’- GGAGCGAGATCCCTCCAAAAT-3’  Reverse 5’- GGCTGTTGTCATACTTCTCATGG -3’ | |

**Supporting data**


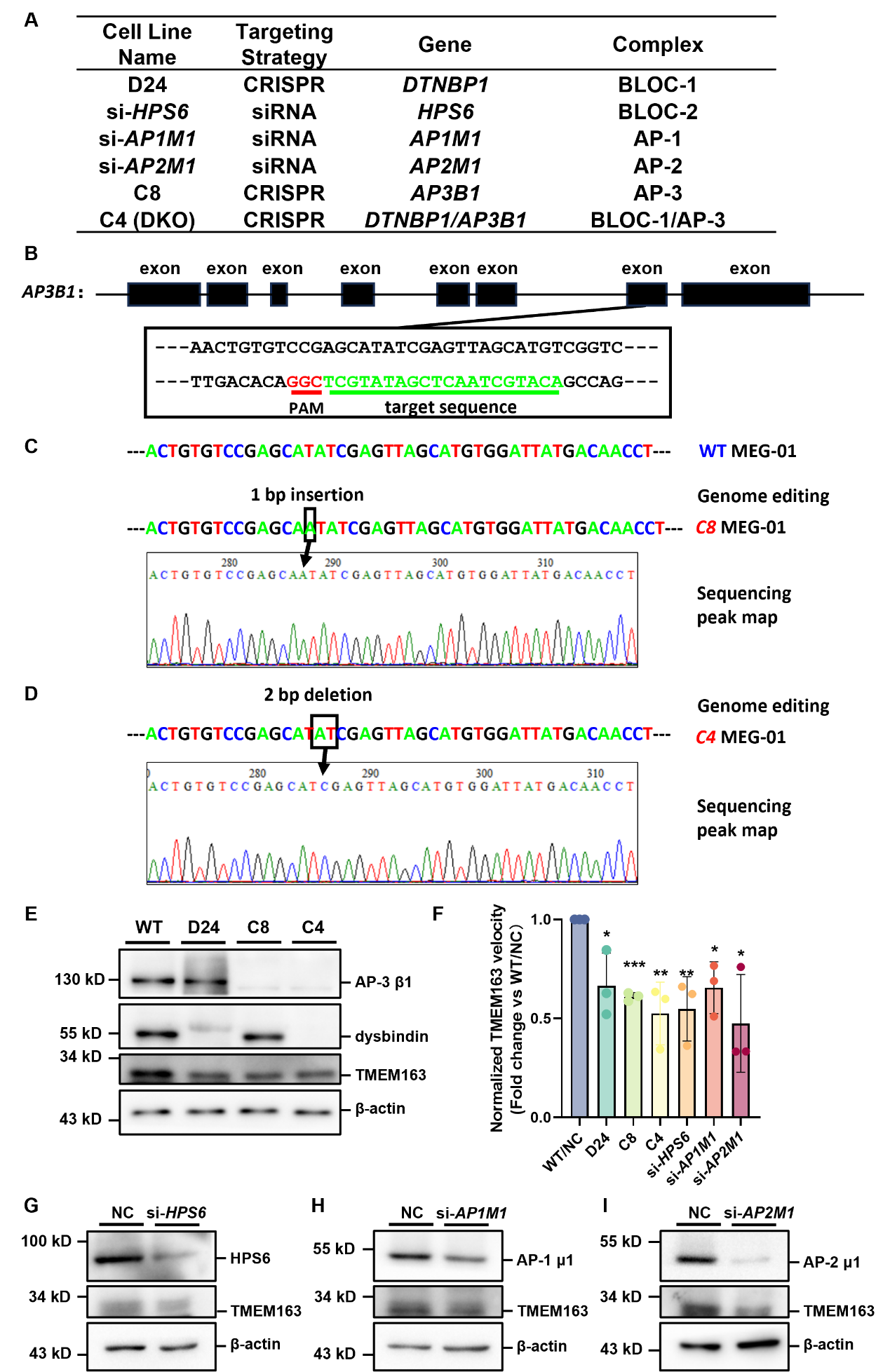


**Figure S1. TMEM163 protein destabilization in megakaryocytic cells caused by deficiencies in BLOC-1, BLOC-2, or AP-complexes.** *A,* list of genes and subunits specifically knocked out or knocked down in each cell line. *B,* strategies for constructing CRISPR/Cas9 stable cell lines by targeting exon 7 of *AP3B1*. *C* and *D,* diagram of genomic editing sequences of *AP3B1-KO* **(**C, *C8***)** and *DTNBP1/AP3B1* *double-KO* (D, *C4*) stable MEG-01 cell lines using CRISPR/Cas9, compared with wild-type (WT) MEG-01. Altered genomic sequences are highlighted with black frames. *E,* immunoblot analysis of WT, *DTNBP1-*KO (*D24*), *AP3B1-*KO (*C8*) and *DTNBP1/AP3B1-*DKO (*C4*) stable cell lines. β-actin serves as a loading control. *F,* statistical data showed the expression of TMEM163 showed significant reduction compared to WT/NC. Each bar represents the mean ± SD, n = 3. Unpaired Student’s *t*-test. * *P* < 0.05; ** *P* < 0.01; *** *P* < 0.001. *G-I,* representative immunoblots of negative control (NC), BLOC-2 knockdown (si-*HPS6*) (G), AP-1 knockdown (si-*AP1M1*) (H), and AP-2 knockdown (si-*AP2M1*) (I), along with corresponding changes in TMEM163 protein expression levels in MEG-01 cells. β-actin serves as a loading control.


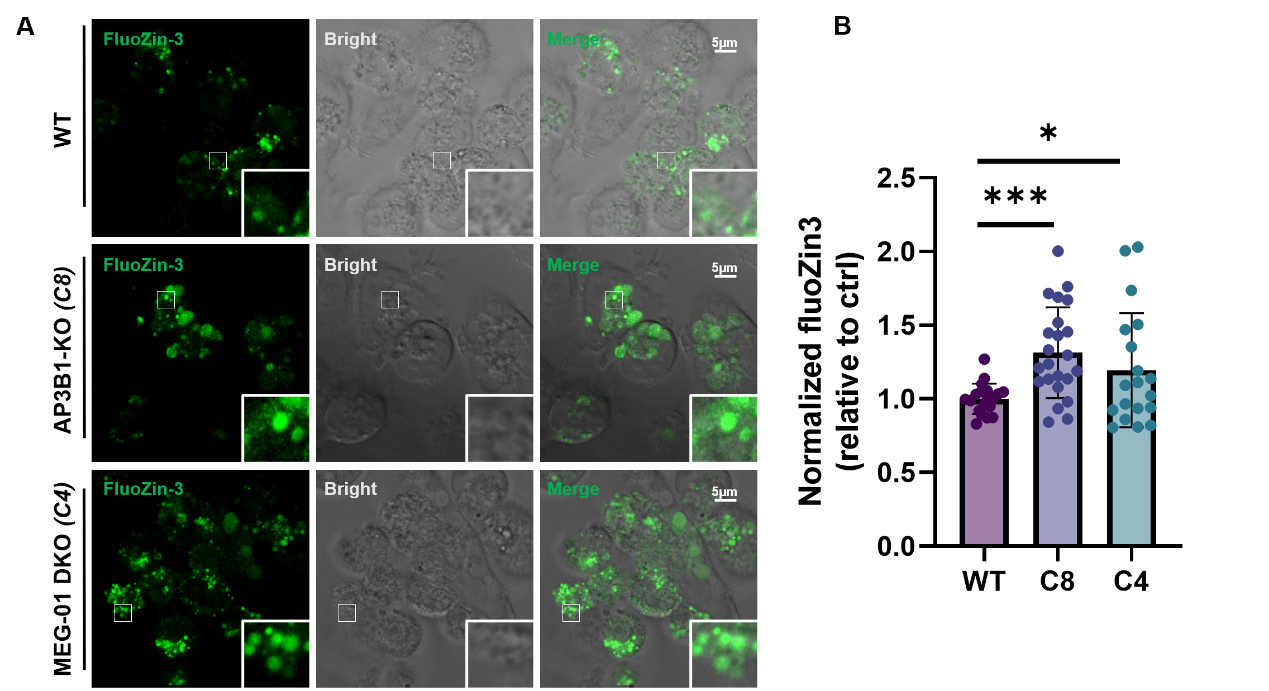


**Figure S2. Accumulated Zn^2+^ in *AP3B1-*KO and *DTNBP1/AP3B1*-DKO MEG-01 cells.** *A-B,* *AP3B1*-KO (*C8*) and *DTNBP1/AP3B1*-DKO (*C4*) MEG-01 cells were treated with 100 nM TPA for 48 hours then incubated with 2 mM Zn^2+^ indicator (FluoZin-3) for 30 minutes at 37°C. The white boxes on the right-bottom represent the intracellular fluorescence intensity of Zn^2+^ which are magnified 4-fold in the insets. Scale bars, 5 μm. The average fluorescence intensity of Zn^2+^ in *C8* and *C4* MEG-01 cells are significantly higher than that in control (WT) cells (WT, 1.000 ± 0.023, n = 19; *C8*, 1.313 ± 0.064, n = 23; *C4*, 1.196 ± 0.088, n = 19).


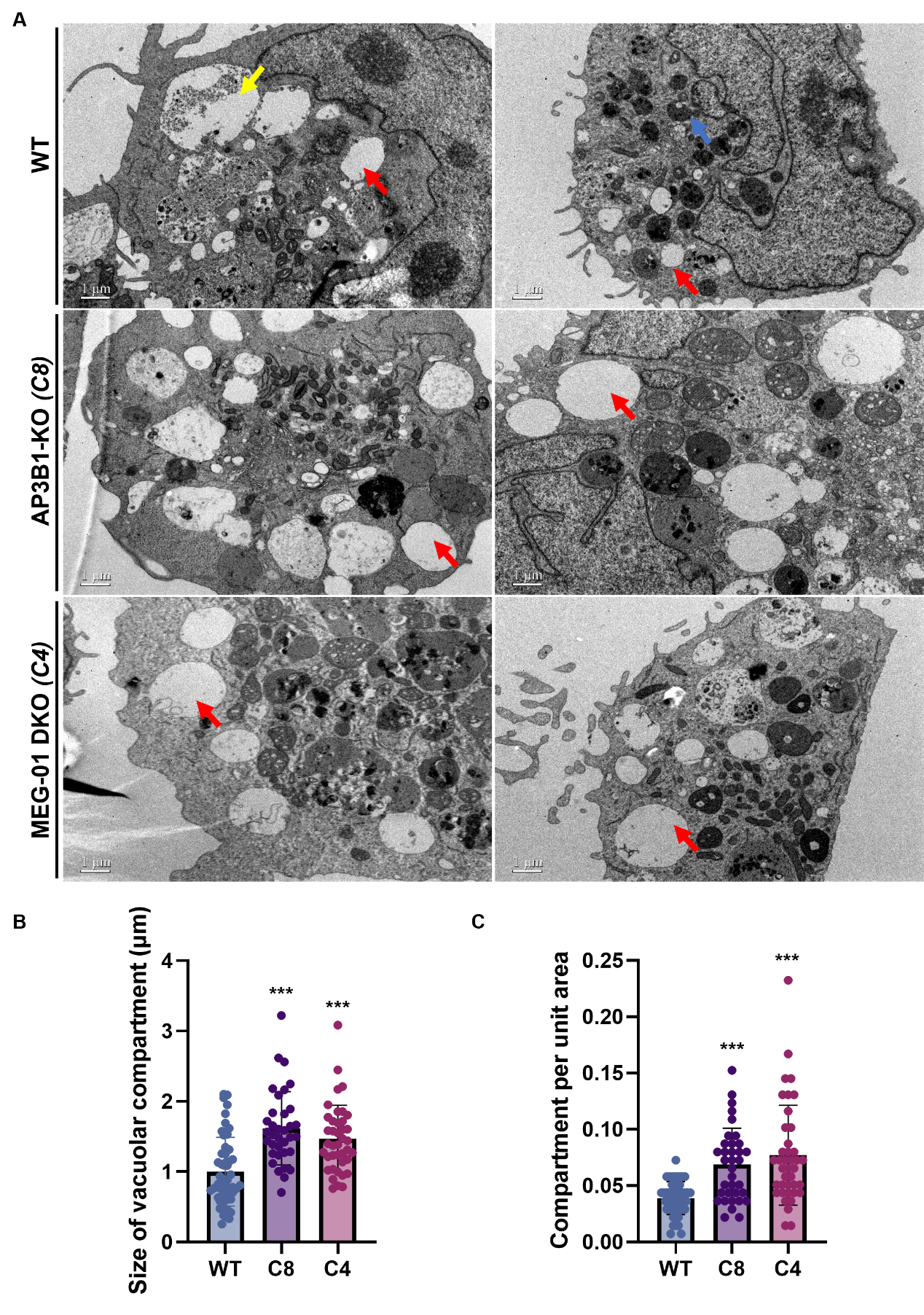


**Figure S3. Ultrastructure of DG precursors in *AP3B1-*KO and *DTNBP1/AP3B1*-DKO MEG-01 cells.** *A-C,* Thin-section TEM pictures of *AP3B1*-KO (*C8*) and *DTNBP1/AP3B1*-DKO (*C4*) MEG-01 cells after treated with 100 nM TPA for 4 days (A). Red arrows represent round membranous organelles resembling EEs with reduced intraluminal contents, yellow arrows represent round membranous organelles resembling LEs with increased intraluminal contents, and blue arrows represent MVB circular membranous organelles. Scale bars, 1 mm. The average number of vacuolar compartment (red arrows shown in panel A) per unit area (μm^2^ ) in *C8* and *C4* MEG-01 cells (B) are significantly higher than that in control (WT) cells (WT, 0.03 ± 0.002, n = 50; *C8*, 0.06 ± 0.005, n = 35; *C4*, 0.07 ± 0.007, n = 40). The average size of the vacuolar compartment (μm^2^) in *C8* and *C4* MEG-01 cells (C) is significantly larger than that in WT cells (WT, 1.000 ± 0.069, n = 50; C8, 1.616 ± 0.088, n = 35; D24, 1.465 ± 0.075, n = 40).


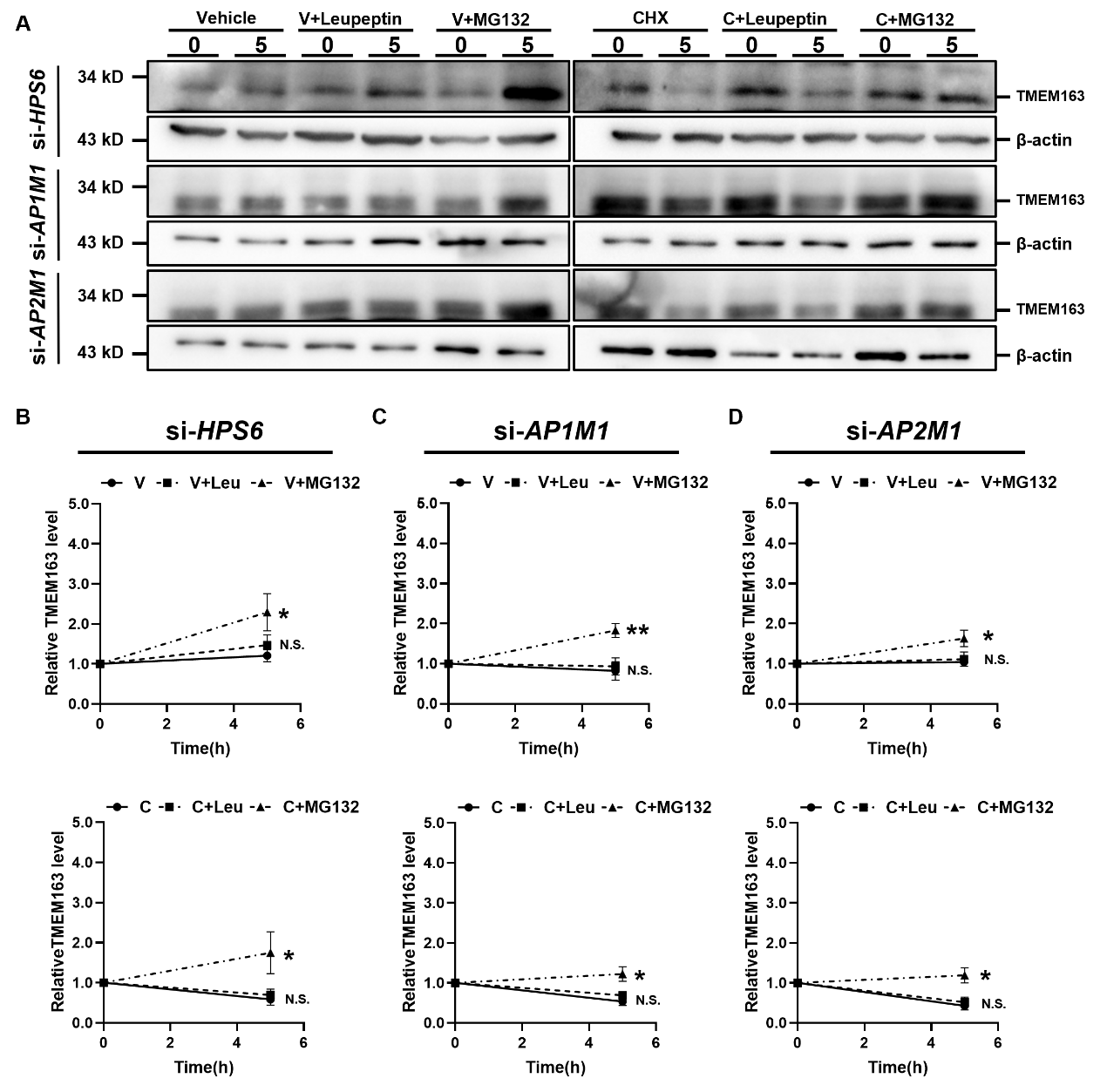


**Figure S4. The degradation of endogenous TMEM163 by knocking down BLOC-2, AP-1 and AP-2.** *A-D,* MEG-01 cells were transfected with BLOC-2-specific siRNA (si-*HPS6*) (B), AP-1 specific siRNA (si-*AP1M1*) (C), AP-2 specific siRNA (si-*AP2M1*) (D) for 24 hours. Cells were collected at 0 and 5 hours after treatment with either DMSO (control) or 20 μM CHX, in combination with 100 μM leupeptin or MG132 as indicated. TMEM163 degradation was analyzed by Western blotting. Solid lines: Vehicle or CHX only; Dashed lines: Vehicle or CHX + leupeptin; Dash-dot lines: Vehicle or CHX + MG132. β-actin serves as a loading control. Statistical significance was determined by paired Student’s *t-*test. Asterisks denote significant differences between groups: **P* < 0.05; ***P* < 0.01; N.S., not significant.


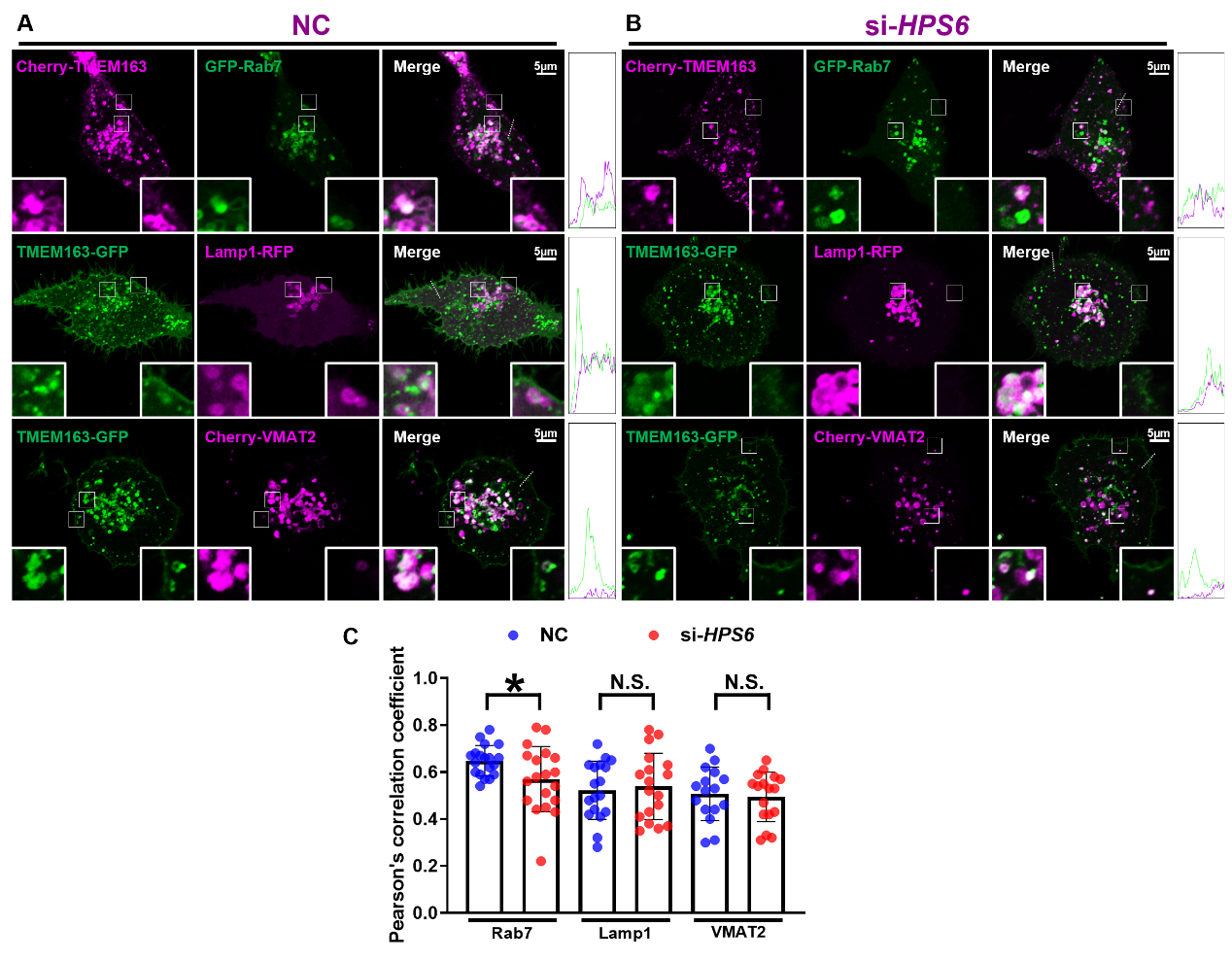


**Figure S5. Live-cell imaging of exogenously expressed TMEM163 in BLOC-2 knockdown cells.** *A-C,* Effect of BLOC-2 knockdown on the subcellular localization of TMEM163. MEG-01 cells were co-transfected with Cherry-TMEM163 and GFP-Rab7, or TMEM163-GFP with Lamp1-RFP/Cherry-VMAT2, along with negative control (NC) (A) or si-*HPS6* (B). After 24 hours, live-cell imaging was performed using confocal microscopy. The white box regions on the right-bottom represents PM localization, and the white box regions on the left-bottom represents the intracellular localization which are magnified 4-fold in the insets. Scale bars: 5 μm. The 5 μm white dashed lines in the merge panel outline regions of PM used for fluorescence intensity quantification across green and magenta channels. Line scan profiles of the corresponding fluorescence intensities are provided in right rectangles. PCC values (C): Rab7 (NC vs si-*HPS6*: 0.64 ± 0.01 vs 0.57 ± 0.03); Lamp1 (NC vs si-*HPS6*: 0.52 ± 0.02 vs 0.53 ± 0.03); VMAT2 (NC vs si-*HPS6*: 0.50 ± 0.02 vs 0.49 ± 0.02). PCC ≥ 0.4 represents colocalization. Data are presented as mean ± SD; 18-20 cells were analyzed per sample. Unpaired Student’s *t*-test was used. **P* < 0.05; N.S., not significant.

**
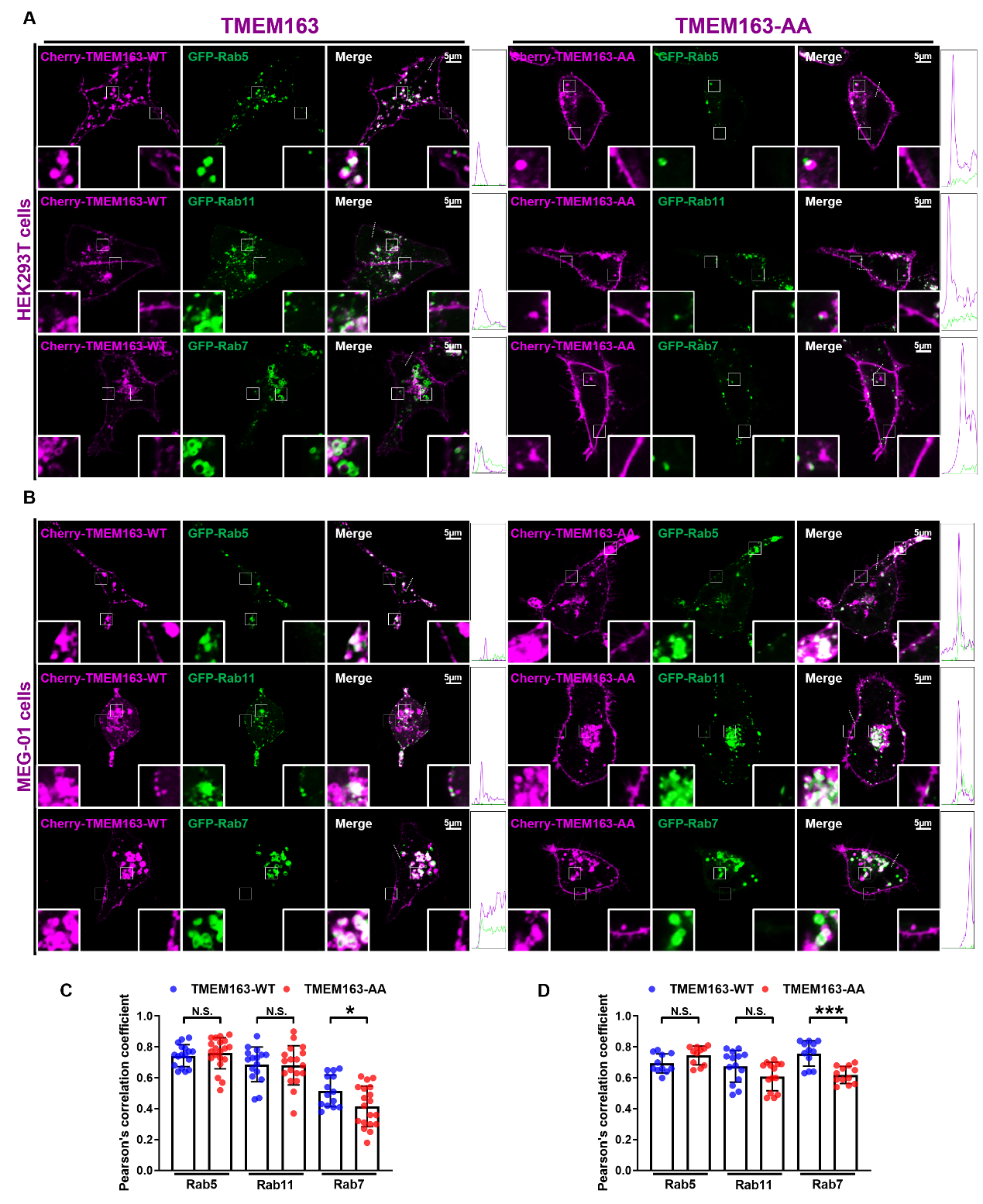
**

**Figure S6. Live-cell imaging of exogenously expressed TMEM163-WT/AA with endosomal markers in HEK293T cells and MEG-01 cells.** *A* and *B,* HEK293T cells (A) and MEG-01 cells (B) were co-transfected with Cherry-TMEM163-WT/Cherry-TMEM163-AA and GFP-Rab5/GFP-Rab11/GFP-Rab7 for 24 hours, respectively. The transfected and stained cells were photographed in living cell dishes. The white box regions on the left represent the intracellular localization, and the white box regions on the right represent the PM localization which are magnified 4-fold in the insets. Scale bars: 5 μm. The 5-μm white dashed lines in the merge panel outline regions of PM used for fluorescence intensity quantification across green and magenta channels. Line scan profiles of the corresponding fluorescence intensities are provided in right rectangles. *C* and *D*, colocalization statistical analysis of Pearson’s correlation coefficient (PCC). HEK293T cells (C): PCC in Rab5 (WT vs AA, 0.74± 0.01 vs 0.75 ± 0.02); PCC in Rab11 (WT vs AA, 0.68 ± 0.02 vs 0.68 ± 0.03); PCC in Rab7 (WT vs AA, 0.51 ± 0.02 vs 0.41 ± 0.03). MEG-01 cells (D): PCC in Rab5 (WT vs AA, 0.69 ± 0.01 vs 0.74 ± 0.01); PCC in Rab11 (WT vs AA, 0.67 ± 0.02 vs 0.61 ± 0.02); PCC in Rab7 (WT vs AA, 0.75 ± 0.02 vs 0.61 ± 0.01). PCC ≥ 0.4 represents colocalization. Data represent mean ± SD across 10-15 cells for each sample. Unpaired Student’s *t*-test. **P* < 0.05; ****P* < 0.001; N.S., not significant.

**
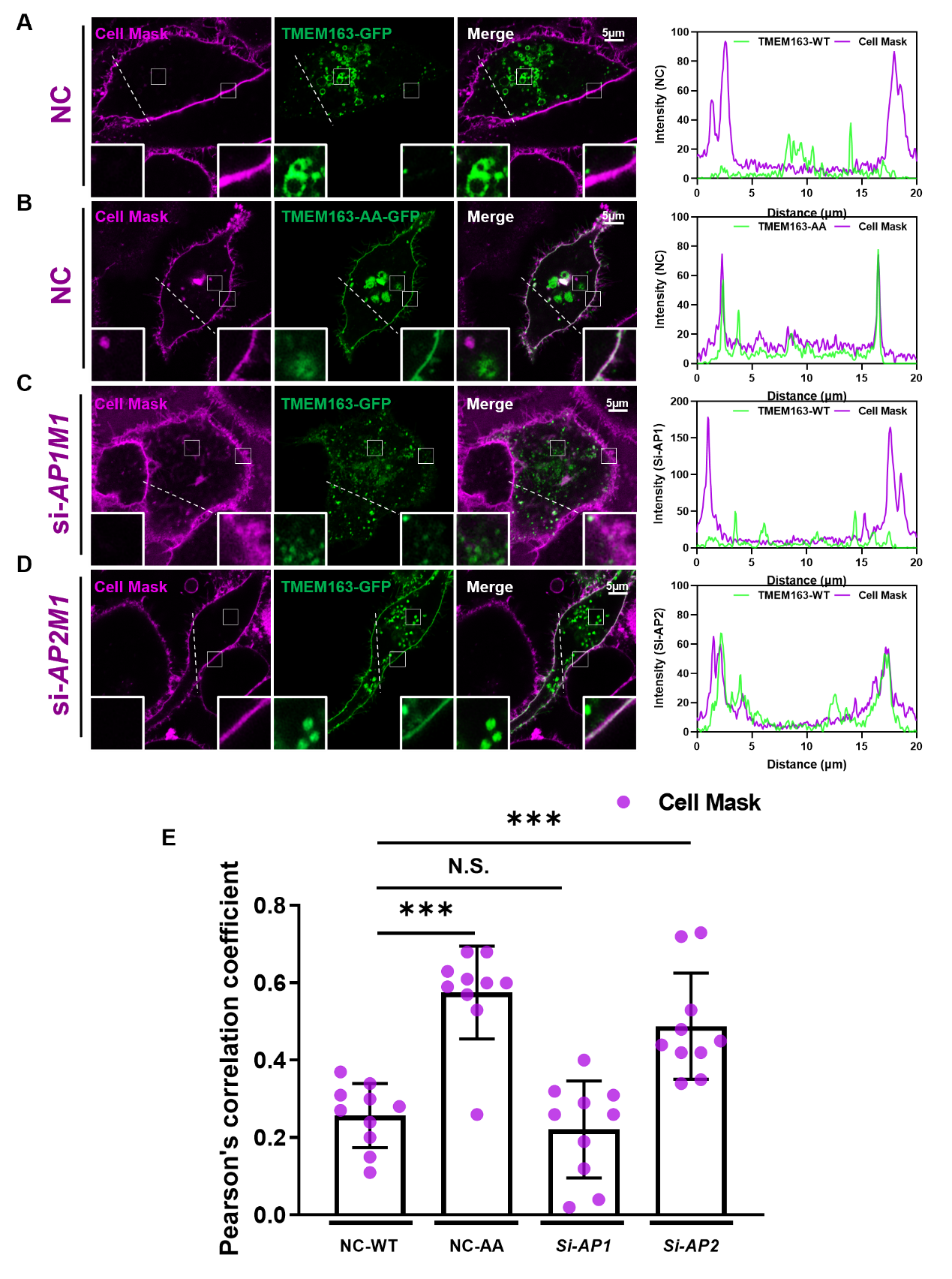
**

**Figure S7. Effect of the AP-1 and AP-2 complex depletion on the subcellular localization of TMEM163.** *A-D,* Subcellular localization of TMEM163 (A, C, D) and the TMEM163-AA mutant (B) under different conditions: negative controls (NC-WT and NC-AA) (A, B), AP-1 knockdown (si-*AP1M1* or si*-AP1*) (C), and AP-2 knockdown (si-*AP2M2* or si*-AP2*) (D). Cells were transfected with TMEM163-GFP for 12 hours, followed by siRNA treatment for 24 hours before imaging. Cell membrane was labeled with Cell Mask stain (10 minutes, 37°C). The white box regions on the right-bottom represents PM localization, and the white box regions on the left-bottom represents the intracellular localization which are magnified 4-fold in the insets. Scale bars: 5 μm. The 20 μm white dashed lines in the merge panel outline regions of interest used for fluorescence intensity quantification across green and magenta channels. Line scan profiles of the corresponding fluorescence intensities are provided in right rectangles. *E*, colocalization statistical analysis of Pearson’s correlation coefficient (PCC). PCC values: Cell Mask (NC-WT/NC-AA/si-AP1/si-AP2, 0.25 ± 0.02/0.57 ± 0.03/0.22 ± 0.03/0.48 ± 0.04). Data are presented as mean ± SD; 10 cells were analyzed per sample. Unpaired Student’s *t*-test was used. ****P* < 0.001; N.S., not significant.
